# One-year and 5-year transition risks between major bleeding, reinfarction, and death following acute myocardial infarction in England and Wales: a population-based cohort study

**DOI:** 10.1093/ehjacc/zuag088

**Published:** 2026-06-24

**Authors:** Sharon A Ayayo, Glen P Martin, Salwa S Zghebi, Vicky P Taxiarchi, Muhammad Rashid, Evangelos Kontopantelis, Mamas A Mamas

**Affiliations:** Faculty of Biology, Medicine and Health, Division of Informatics, Imaging and Data Sciences, The University of Manchester, Core Technology Facility, 46 Grafton Street, Manchester, M13 9NT, United Kingdom; Faculty of Biology, Medicine and Health, Division of Informatics, Imaging and Data Sciences, The University of Manchester, Core Technology Facility, 46 Grafton Street, Manchester, M13 9NT, United Kingdom; Faculty of Biology, Medicine and Health, Division of Population Health, Health Services Research and Primary Care, The University of Manchester, Manchester, United Kingdom; Faculty of Biology, Medicine and Health, Centre for Women’s Mental Health, Division of Psychology and Mental Health, The University of Manchester, Manchester, United Kingdom; Department of Cardiovascular Sciences, National Institute for Health and Care Research (NIHR) Leicester Cardiovascular Biomedical Research Unit, Glenfield Hospital, Leicester, United Kingdom; Keele Cardiovascular Research Group, Keele University, Stoke on Trent, United Kingdom; Faculty of Biology, Medicine and Health, Division of Informatics, Imaging and Data Sciences, The University of Manchester, Core Technology Facility, 46 Grafton Street, Manchester, M13 9NT, United Kingdom; Keele Cardiovascular Research Group, Keele University, Stoke on Trent, United Kingdom; National Institute for Health and Care Research (NIHR) Birmingham Biomedical Research Centre, Birmingham, United Kingdom

**Keywords:** Acute myocardial infarction, MINAP, Bleeding, Reinfarction, Mortality, Multistate modelling

## Abstract

**Aims:**

There is limited data on the long-term transition risks between major bleeding, reinfarction, and death following acute myocardial infarction (AMI). We examined 1-year and 5-year transition risks of major bleeding, reinfarction, and mortality post-AMI.

**Methods and results:**

We analysed national registry data of AMI patients across England and Wales between 2005 and 2021. Multistate modelling was used to estimate the transition-specific adjusted incidence rates (IRs) across the three outcomes using a six-state model with nine transitions. Out of 837 384 patients included, 19.8% had bleeding, 8.1% reinfarction, and 20.6% death as their first event post- AMI admission. Of those with bleeding, 39.4% died while 49.9% of those with reinfarction died. Overall, females had higher rates of transitioning to any of the five states than males. A reference female patient with median age (76.3 years), at 1-year had an IR of 6.7 (95% CI 6.5–6.9) ×100 person-years (PY) for AMI admission → bleeding transition, 0.2 (95% CI 0.2–0.2) ×100PY for AMI admission → reinfarction, 43.5 (95% CI 41.9–45.2) ×100PY for AMI admission → death, 22.2 (95% CI 20.9–23.5) ×100PY for bleeding → death, and 41.9 (95% CI 38.1–46.0) ×100PY for reinfarction → death. At 5-year, the IRs decreased to 2.1 (95% CI 2.0–2.2) ×100PY, 0.04 (95% CI 0.04–0.05) ×100PY, 17.5 (95% CI 16.9–18.2) ×100PY, 9.7 (95% CI 9.2–10.3) ×100PY, and 17.6 (95% CI 16.0–19.4) ×100PY, respectively. A reference male patient with median age (67.1 years) at 1-year had an IR of 5.2 (95% CI 5.0–5.4) ×100PY for AMI admission → bleeding transition, 0.2 (95% CI 0.2–0.2) ×100PY for AMI admission→ reinfarction, 23.7 (95% CI 22.8–24.6) ×100PY for AMI admission → death, 14.5 (95% CI 13.6–15.4) ×100PY for bleeding → death, and 25.9 (95% CI 23.8–28.3) ×100PY for reinfarction → death. Similarly, 5-year IRs decreased to 1.6 (95% CI 1.6–1.7) ×100PY, 0.04 (95% CI 0.04–0.04) ×100PY, 9.5 (95% CI 9.2–9.9) ×100PY, 6.3 (95% CI 5.9–6.8) ×100PY, and 10.9 (95% CI 10.0–11.9) ×100PY, respectively. Also, those with diabetes and white patients had higher transition rates than those without diabetes and black patients.

**Conclusion:**

In this national AMI cohort, 5-year direct death and bleeding events were more frequent than reinfarction, and the highest mortality occurred in women after AMI admission and in men following reinfarction. These transition patterns quantify the substantial absolute risks patients face post-AMI and highlight important sex-specific differences in adverse outcomes.

## Structured Graphical Abstract

**Structured Graphical Abstract:**
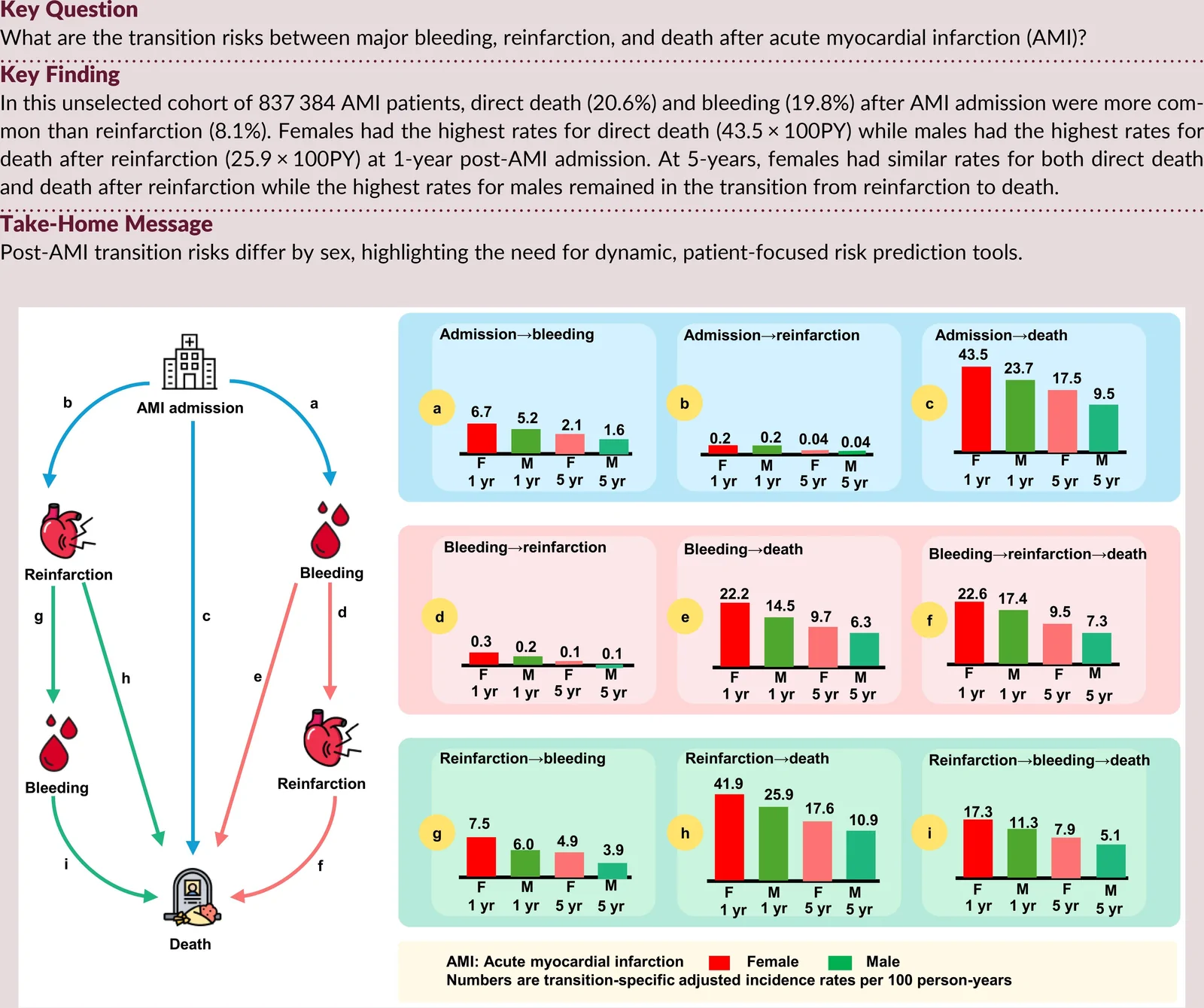

## Introduction

Acute myocardial infarction (AMI) remains a major global health burden, despite significant advances in its diagnosis and management.^1^ Following an initial AMI event, patients remain at a substantial risk for recurrent cardiovascular events and complications, including major bleeding, reinfarction, and death, which are among the most common complications.^1,2^ Previous studies reported incidence rates (IRs) of 8.6% for major bleeding during a median follow-up period of 4.6 years,^3^ 1.8% at 30 days to 6.9% at 3 years for reinfarction in the HORIZONS-AMI trial,^4^ and 37.8% for all-cause mortality at 9 years.^5^ These events are interrelated, with each potentially influencing the risk of subsequent events. For example, bleeding complications may necessitate the discontinuation of antiplatelet therapy, thereby increasing the risk of reinfarction. On the other hand, reinfarction can necessitate the escalation of antithrombotic treatment, thereby elevating bleeding risk.^6,7^ Prior studies report that both bleeding and reinfarction are associated with an increased risk of mortality.^8,9^ This interdependent relationship complicates clinical decision-making, especially in patients with coexisting ischaemic and bleeding risks. Understanding how these complications relate to one another is critical for optimizing post-AMI care and improving outcomes.

Despite the clinical importance of these interrelated outcomes, no study to the best of our knowledge has examined the temporal pathways between bleeding, reinfarction, and death after AMI. Most existing evidence focuses on each outcome separately or compares how strongly bleeding and reinfarction predict future events without exploring the transition risks between them. Therefore, the aim of our study is to explore the transition risks between bleeding, reinfarction, and death after AMI, and the sequence in which they occur, using a real-world national AMI registry in England and Wales.

## Methods

### Study design

Retrospective observational cohort analysis of prospectively collected data.

### Data resources and study population

We used the Myocardial Ischaemia National Audit Project (MINAP) registry, linked with Hospital Episode Statistics (HES) and Office for National Statistics (ONS) mortality data. MINAP is a national registry recording all AMI admissions [ST-elevated MI (STEMI) and non-ST-elevated MI (NSTEMI)] across 230 acute hospitals in England and Wales, capturing 130 variables, including demographics, medical history, treatments, and outcomes.^10^ Data are pseudonymized and submitted to the National Institute of Cardiovascular Outcomes Research (NICOR). HES Admitted Patient Care (HES APC) is a registry composed of data that are collected on all inpatient admissions to the National Health Service (NHS) hospitals in England, covering demographics and clinical data.^11^ As HES APC captures all hospital admissions, the absence of an event provides strong evidence that the event did not occur within the hospital setting. The ONS mortality data provides accurate information on death dates and causes, since death registration is mandatory in the UK.^12^

All adult patients aged 18–100 years admitted with AMI between January 2005 and December 2018 in England and Wales were included in the analysis. Patients were followed up until July 2021. Only the first admission for AMI per patient was retained to define an index cohort of unique patients who were then followed for bleeding, reinfarction, or death. Those with missing data on age and sex were excluded for data quality purposes. (Supplementary material online, *Figure S1* gives the full inclusion/exclusion criteria).

### Outcomes and covariates

Outcomes were the first occurrence of any major bleeding, reinfarction, and death within 5 years post-AMI admission. Major bleeding was defined as any clinically actionable bleeding event that necessitated hospitalization or resulted in death either during the hospital stay or following discharge. Reinfarction was defined as any subsequent AMI of all types occurring within 5 years post-AMI admission. The index AMI, obtained from MINAP, reflects predominantly Type 1 MI as MINAP primarily captures spontaneous AMI. Death was defined as all-cause death that occurred within 5 years post-AMI admission. Bleeding and reinfarction outcomes were identified from the linked HES APC database using ICD-10 codes (see Supplementary material online, *Table S1*), and date of death was obtained from the ONS mortality registry. Patients were followed up until death, 5 years from prevalent AMI admission date or the end of data extraction (15 July 2021), whichever occurred first. Only the first occurrence of bleeding and reinfarction events were considered in the analysis. Outcomes were recorded at the day level and not at the hour or minute level; therefore, we could not differentiate the order of events that happened on the same day. Consequently, we excluded simultaneous transitions of bleeding and reinfarction as they were rare [3125 (0.4%)] and do not inform transitions between these two states. However, when bleeding or reinfarction occurred on the same day as death, then death was assumed to occur last, as it represents the terminal event in the clinical pathway.

Covariates obtained from MINAP included demographics (age, sex, ethnicity), medical history [previous AMI, previous angina, previous coronary artery bypass grafting (CABG), previous percutaneous coronary intervention (PCI), family history of coronary heart disease (CHD), hypertension, hypercholesterolaemia, diabetes, asthma/chronic obstructive pulmonary disease, cerebrovascular disease, peripheral vascular disease (PVD), chronic renal failure, heart failure, smoking status], medications and interventions during hospital admission (P2Y12 inhibitors, aspirin, warfarin, glycoprotein IIb/IIIa inhibitors, statins, PCI, CABG), and examinations on admission (heart rate, systolic blood pressure).

### Statistical analysis

Baseline covariates, stratified by whether a patient had each event at any point in follow-up (i.e. bleeding at any time, reinfarction at any time, and all-cause mortality at any time), were summarized descriptively by means and standard deviations (SD), or medians and interquartile ranges (IQR) for continuous variables as appropriate, and by frequencies and percentages for categorical variables. Groups were compared by Pearson’s *χ*^2^ test for categorical variables and *t*-test or Wilcoxon rank-sum test for continuous variables. For each covariate, the level of data missingness was also reported as numbers and percentages, by year of AMI admission (see Supplementary material online, *Table S2*).

To account for missing data in the inferential analyses, 10 imputed datasets were generated using multiple imputation by chained equations (MICE), and the imputation model included all key variables included in the analytical model as well as the outcomes. Nelson–Aalen (NA) estimators of the survival times for each outcome were included in the imputation model together with the event indicators.^13^ Due to computational complexity, we did not combine estimates across all imputations as per Rubin’s rules, but we used the first imputation for the primary analysis, and three imputations were randomly selected for sensitivity analyses. Continuous variables were imputed using predictive mean matching with five nearest neighbours while binary variables using logistic regression and categorical variables using multinomial/ordered logistic regression.

Multistate modelling^14^ based on the semi-Markov assumption was used to estimate transition-specific incidence rates across the three outcomes by adopting a six-state model with nine transitions between states (*Figure 1*). The semi-Markov assumption allows the risk of transitioning to the next state to depend not only on the current state but also on the time spent in that state, providing a more clinically realistic framework. The six states are admission for AMI (no events), direct bleeding, direct reinfarction, bleeding before reinfarction, reinfarction before bleeding as transient states, and death as the absorbing state. Transitions back to the previous state were not considered, as they were not of primary interest in this study. Patient follow-up initiated at the first AMI-related hospital admission, which defined the index date. Transition-specific incidence rates [per 100 person-years (PY)] and 95% confidence intervals were estimated as a function of time (per year since index date) for each combination of sex, ethnicity, diabetes status, and sex-ethnicity-specific median age (67.8, 62.0, 76.7, and 71.1 years for white males, black males, white females, and black females, respectively) covariate patterns (a reference patient), using separate Weibull models, which are appropriate parametric survival models for time-to-event data, and displayed graphically. Additionally, transition rates for other ages (50, 60, 70, and 80 years) were estimated and displayed in heat maps and forest plots. The Weibull models were fitted via the *merlin*^15^ command in Stata, adjusting for covariates described in the covariates section above and accounting for left truncation. The predicted transition-specific incidence rates were estimated via the *predictms*^16^ post-estimation command of the multistate package in Stata. In all models, a clock reset approach timescale was used, whereby transitions were modelled using the time since entry into each state, rather than time since entering the initial admission for AMI state. Transition-specific incidence rates measure the risk of how quickly patients move from one state to another at a given time and how this risk changes over the follow-up period. In other words, patients with higher rates are more likely to move into the subsequent health state sooner than those with lower rates.

**Figure 1:**
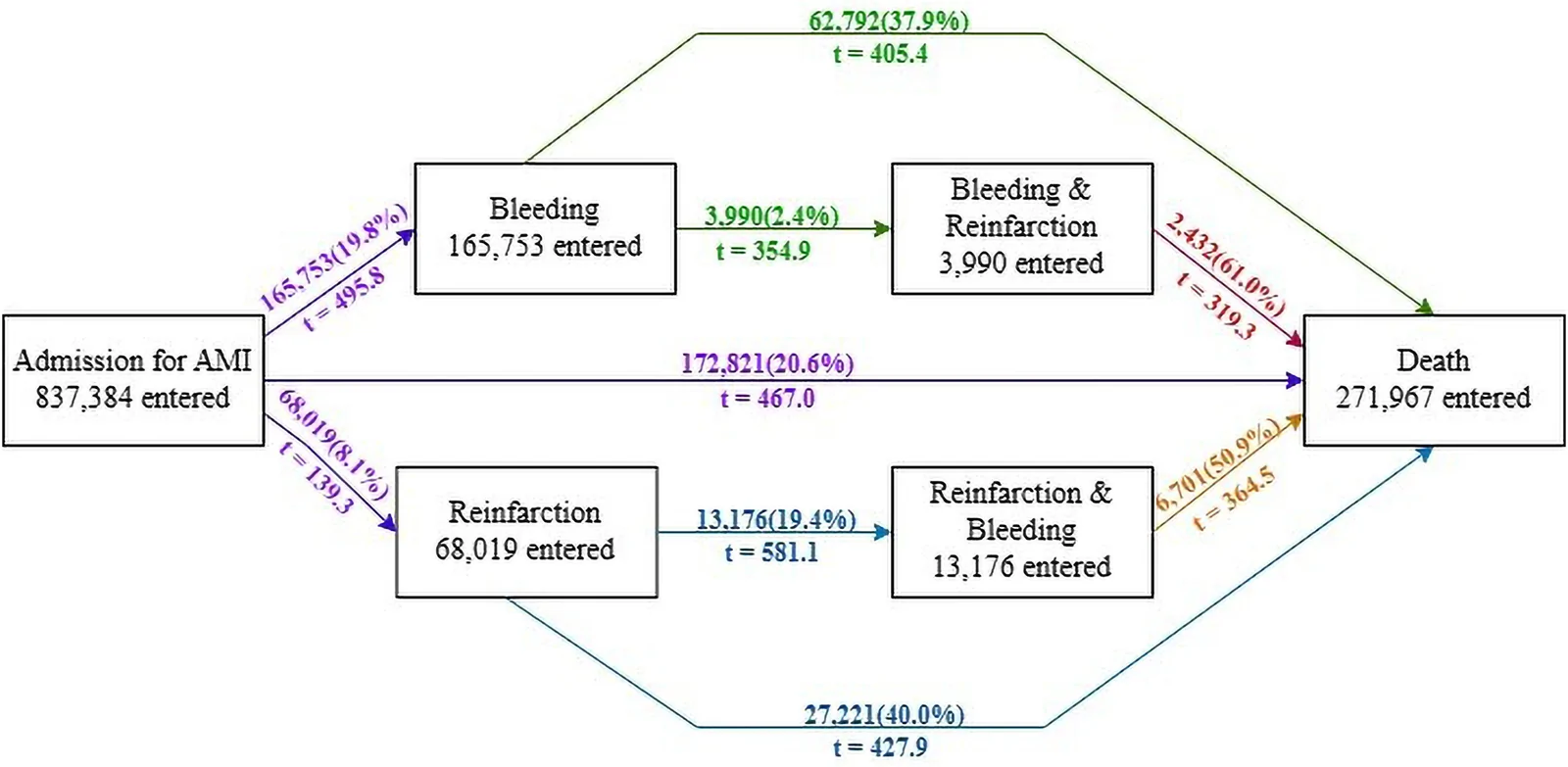
State space diagram showing observed transitions from AMI admission to bleeding, reinfarction, and death for all patients within 5 years post-AMI admission. Number entered shows the number of patients entering each state; number (%) along the arrows shows the number and percentage of patients leaving one state and entering the other; *t* shows the time to transition from one state to another (in days). Numbers of patients that entered each state are used as the denominators for the percentages relating to that state. Bleeding & Reinfarction state mean bleeding occurred before reinfarction, and Reinfarction & Bleeding state mean reinfarction occurred before bleeding.

All models were fitted using Stata/MP v17,^17^ and graphs produced using R version 4.2.1.^18^ All *P*-values were two-sided, and a 5% level of significance was used.

## Results

A total of 837 384 patients were included in the analysis. Missing data for the key variables in the imputation model ranged from 2.4% in ethnicity to 25.8% in family history of CHD variables (see Supplementary material online, *Table S2*).

At any time within 5 years post-AMI admission, 178 929 (21.4%) patients had bleeding, 72 009 (8.6%) reinfarction, and 271 967 (32.5%) died from any cause. As summarized in *Table 1*, patients who experienced bleeding, reinfarction, or death were generally older and had a higher comorbidity burden like hypertension, diabetes, hypercholesterolaemia, previous AMI, PVD, cerebrovascular disease, and renal failure compared with those who remained free of these outcomes.

**Table 1:** Patient characteristics stratified by bleeding at any time within 5-year follow-up, reinfarction at any time within 5-year, and all-cause mortality at any time within 5-year follow-up

	Bleeding		Reinfarction		All-cause mortality	
Variable	No	Yes	No	Yes	No	Yes
*n*	658 455	178 929	765 375	72 009	565 417	271 967
Age at AMI admission, median (IQR)	68.7 (57.7, 79.3)	74.8 (65.2, 82.3)	69.7 (58.6, 79.8)	75.4 (65.2, 83.0)	64.8 (55.4, 74.1)	80.8 (73.0, 86.6)
Systolic blood pressure, median (IQR)	138.0 (120.0, 156.0)	138.0 (120.0, 157.0)	138.0 (120.0, 156.0)	138.0 (120.0, 158.0)	140.0 (121.0, 158.0)	134.0 (114.0, 154.0)
Heart rate on AMI admission, median (IQR)	78.0 (66.0, 91.0)	79.0 (66.0, 92.0)	78.0 (66.0, 91.0)	80.0 (67.0, 95.0)	75.0 (65.0, 88.0)	84.0 (70.0, 100.0)
Sex						
Male	439 254 (66.7%)	112 833 (63.1%)	504 495 (65.9%)	47 592 (66.1%)	397 257 (70.3%)	154 830 (56.9%)
Female	219 201 (33.3%)	66 096 (36.9%)	260 880 (34.1%)	24 417 (33.9%)	168 160 (29.7%)	117 137 (43.1%)
Ethnicity						
White	579 982 (88.1%)	164 090 (91.7%)	678 540 (88.7%)	65 532 (91.0%)	498 132 (88.1%)	245 940 (90.4%)
Black	6589 (1.0%)	1743 (1.0%)	7830 (1.0%)	502 (0.7%)	6107 (1.1%)	2225 (0.8%)
Asian	38 727 (5.9%)	8017 (4.5%)	42 986 (5.6%)	3758 (5.2%)	37 057 (6.6%)	9687 (3.6%)
Mixed	2248 (0.3%)	510 (0.3%)	2583 (0.3%)	175 (0.2%)	2200 (0.4%)	558 (0.2%)
Other	12 563 (1.9%)	2765 (1.5%)	14 642 (1.9%)	686 (1.0%)	12 128 (2.1%)	3200 (1.2%)
Previous AMI	101 139 (15.4%)	33 659 (18.8%)	93 786 (12.3%)	41 012 (57.0%)	70 678 (12.5%)	64 120 (23.6%)
Hypertension	293 320 (44.5%)	93 385 (52.2%)	347 646 (45.4%)	39 059 (54.2%)	244 298 (43.2%)	142 407 (52.4%)
Hypercholesterolaemia	189 043 (28.7%)	55 128 (30.8%)	217 213 (28.4%)	26 958 (37.4%)	173 549 (30.7%)	70 622 (26.0%)
Asthma/chronic obstructive pulmonary disease	85 489 (13.0%)	29 903 (16.7%)	103 785 (13.6%)	11 607 (16.1%)	62 691 (11.1%)	52 701 (19.4%)
Peripheral vascular disease	23 767 (3.6%)	8691 (4.9%)	27 788 (3.6%)	4670 (6.5%)	14 458 (2.6%)	18 000 (6.6%)
Cerebrovascular disease	45 756 (6.9%)	16 637 (9.3%)	54 087 (7.1%)	8306 (11.5%)	25 518 (4.5%)	36 875 (13.6%)
Renal failure	30 146 (4.6%)	12 808 (7.2%)	37 546 (4.9%)	5408 (7.5%)	13 293 (2.4%)	29 661 (10.9%)
Smoking status						
Never smoked	166 061 (25.2%)	45 407 (25.4%)	195 489 (25.5%)	15 979 (22.2%)	144 317 (25.5%)	67 151 (24.7%)
Ex-smoker	195 609 (29.7%)	63 263 (35.4%)	231 602 (30.3%)	27 270 (37.9%)	165 294 (29.2%)	93 578 (34.4%)
Current smoker	177 405 (26.9%)	36 737 (20.5%)	198 958 (26.0%)	15 184 (21.1%)	172 489 (30.5%)	41 653 (15.3%)
Currently non-smoker but smoking history unknown	68 293 (10.4%)	20 017 (11.2%)	80 691 (10.5%)	7619 (10.6%)	50 482 (8.9%)	37 828 (13.9%)
Diabetes	127 808 (19.4%)	40 556 (22.7%)	148 591 (19.4%)	19 773 (27.5%)	95 874 (17.0%)	72 490 (26.7%)
Previous PCI	41 351 (6.3%)	14 706 (8.2%)	46 009 (6.0%)	10 048 (14.0%)	39 012 (6.9%)	17 045 (6.3%)
Previous CABG	31 263 (4.7%)	12 455 (7.0%)	35 076 (4.6%)	8642 (12.0%)	24 331 (4.3%)	19 387 (7.1%)
Previous angina	133 357 (20.3%)	47 523 (26.6%)	146 534 (19.1%)	34 346 (47.7%)	99 980 (17.7%)	80 900 (29.7%)
Family history of CHD	157 896 (24.0%)	35 362 (19.8%)	178 139 (23.3%)	15 119 (21.0%)	161 513 (28.6%)	31 745 (11.7%)
Heart failure	29 658 (4.5%)	10 306 (5.8%)	33 821 (4.4%)	6143 (8.5%)	11 635 (2.1%)	28 329 (10.4%)

In terms of management strategies, P2Y12 inhibitors, warfarin, and statins were used more frequently in patients with bleeding than those without. P2Y12 inhibitors, glycoprotein inhibitors, PCI, and CABG were more likely to be used in patients without reinfarction than those with reinfarction. Those who died were more likely to use warfarin while those who did not die were more likely to use P2Y12 inhibitors, aspirin, statins, glycoprotein inhibitors, PCI, and CABG (see Supplementary material online, *Table S3*). Less than half of the study population underwent coronary revascularization as revascularization rates reflect the real-world case mix across the 2005–21 period, including older, multimorbid, and conservatively managed NSTEMI patients.

Of all 837 384 patients at risk of transitioning to any state from the index date, 165 753(19.8%) transitioned directly to bleeding after an average time of 495.8 days, 68 019(8.1%) transitioned directly to reinfarction after 139.3 days on average, and 172 821(20.6%) transitioned directly to death after 467.0 days on average. Of the 165 753 patients who experienced bleeding as their first event after admission for AMI, 3990(2.4%) progressed to reinfarction after 354.9 days on average, out of which 2432(61.0%) died afterwards after 319.3 days on average, while 62 792(37.9%) died after bleeding without a reinfarction after 405.4 days on average. Of the 68 019 who experienced reinfarction as their first event after admission for AMI, 13 176(19.4%) moved to bleeding state after 581.1 days on average, out of which 6701(50.9%) subsequently died after 364.5 days on average, while 27 221(40.0%) entered death state from reinfarction state without bleeding after 427.9 days on average (*Figure 1*).

*Figures 2* and *3* show heat maps of adjusted incidence rates across all transitions for the different patient populations. Complementary forest plots of the same absolute rate estimates for each transition are provided in Supplementary material online, *Figures S2*–*S13*. Transition rates increased with increasing age for all transitions, with the transition from AMI admission to death in white males aged 80 years with diabetes having the highest transition rate in year one post-AMI admission. However, the rates for all transitions and ages decreased over the follow-up period to about half in Year 5 post-AMI admission.

**Figure 2:**
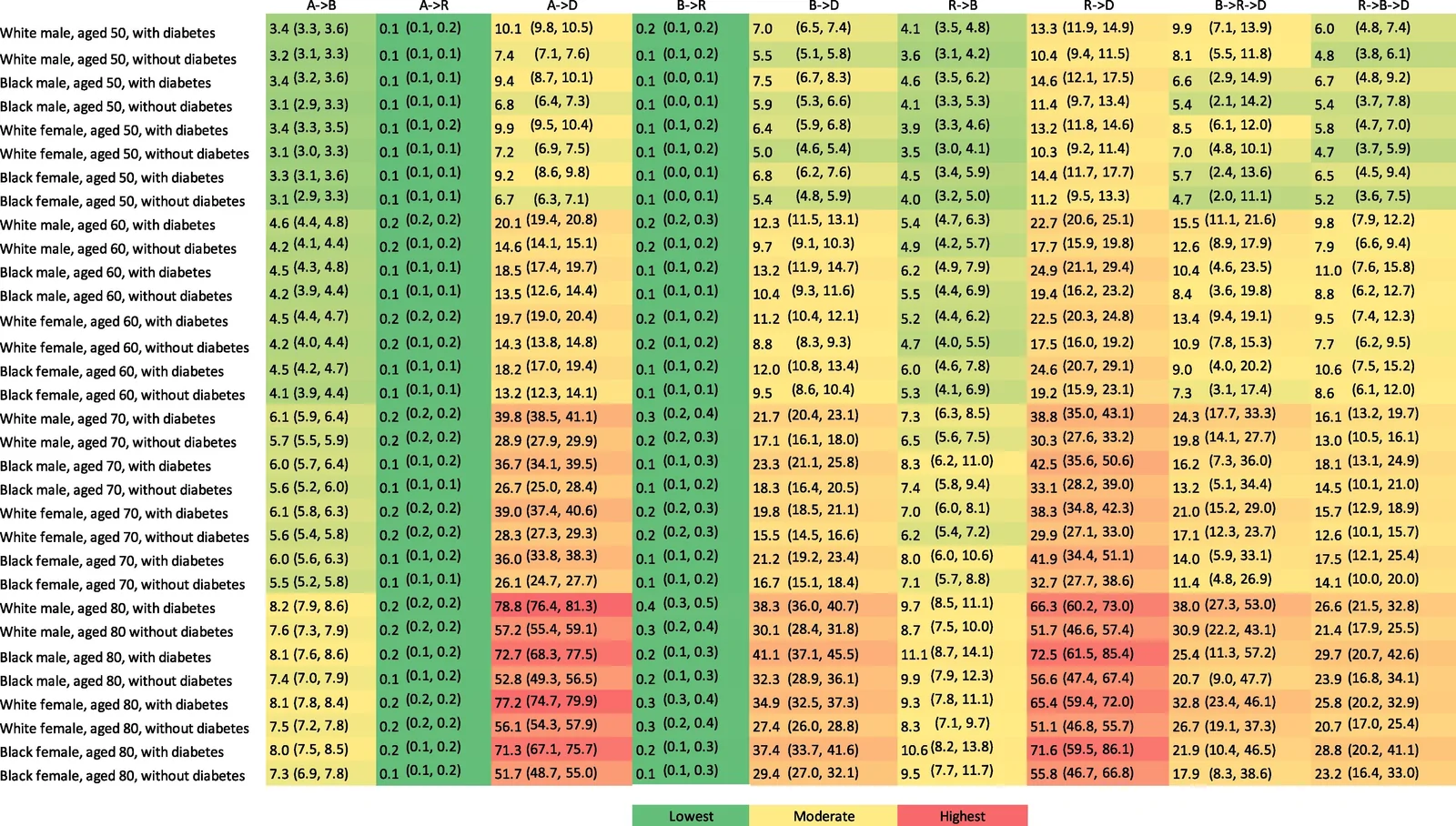
Heat map showing the transition-specific adjusted incidence rates (95% CI) for ages 50, 60, 70, and 80 in Year 1 post-AMI admission. R, reinfarction; D, death; A, admission for AMI; B, bleeding.

**Figure 3:**
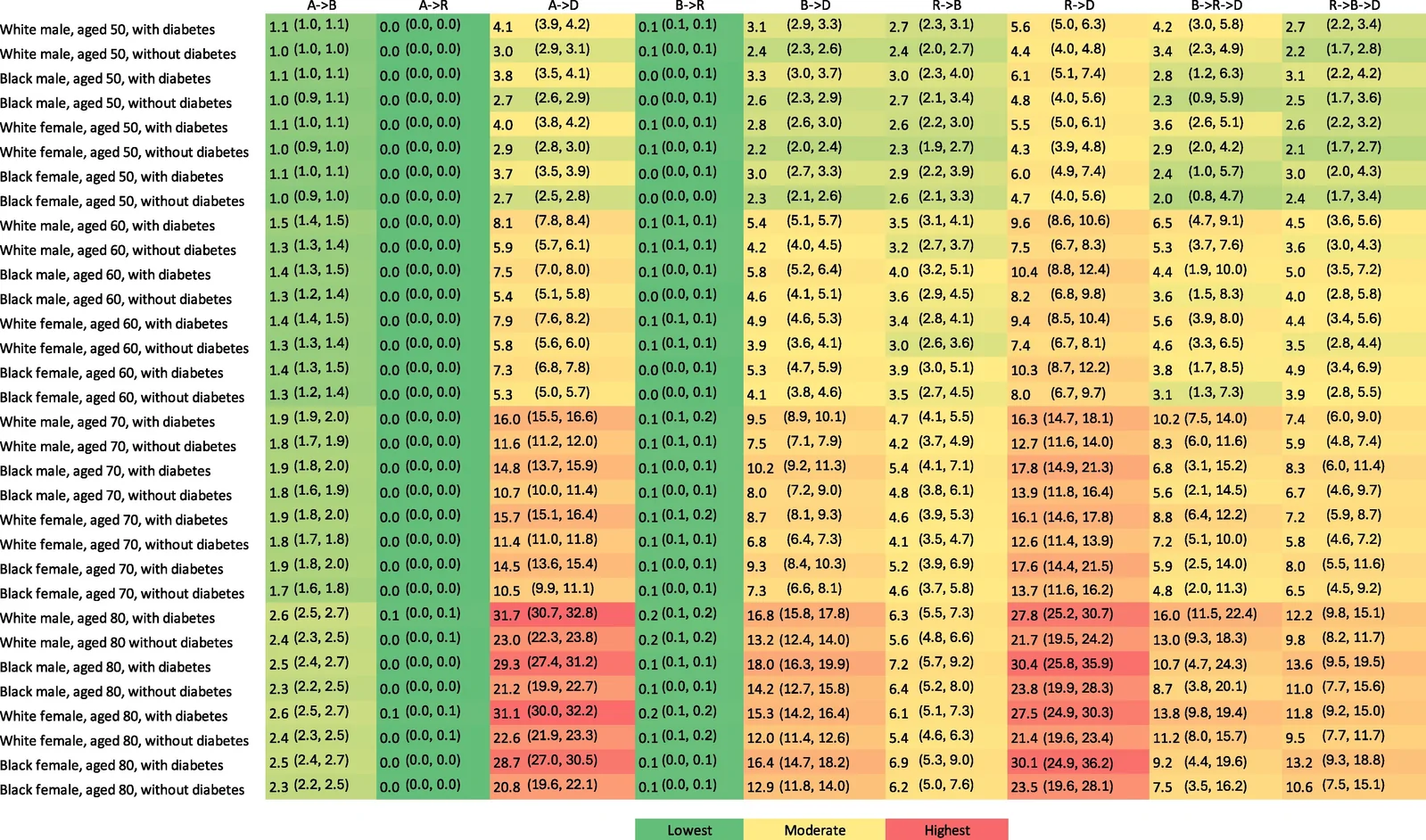
Heat map showing the transition-specific adjusted incidence rates (95% CI) for ages 50, 60, 70, and 80 in Year 5 post-AMI admission. R, reinfarction; D, death; A, admission for AMI; B, bleeding.

Overall, females had higher incidence rates of transitioning to any of the five states after AMI admission than males. For a reference white female patient with the median age of 76.7 years with diabetes, the rate of direct death was the highest in Year 1 post-AMI admission, and this reduced over the 5-year follow-up time (IR: 61.6; 95% CI 59.5–63.9 ×100PY in Year 1 to IR: 24.8; 95% CI 23.9–25.7 ×100PY in Year 5). However, for a reference white male patient with the median age of 67.8 years with diabetes, the rates of both direct transition to death and transitioning to death after reinfarction were similar and the highest in Year 1 post-AMI admission, and this reduced over follow-up time (IR: 34.2; 95% CI 33.0–35.5 ×100PY in Year 1 to IR: 13.8; 95% CI 13.3–14.3 ×100PY in Year 5 for direct death, and IR: 34.5; 95% CI 31.3–38.0 ×100PY in Year 1 to IR: 14.5; 95% CI 13.1–16.0 ×100PY in Year 5 for death after a reinfarction) (*Figure 4*). Those without diabetes had lower rates than those with diabetes in both sexes (*Figure 5*). Conversely, the rates for any transition were higher for males than females of the same age (*Figure 2*).

**Figure 4:**
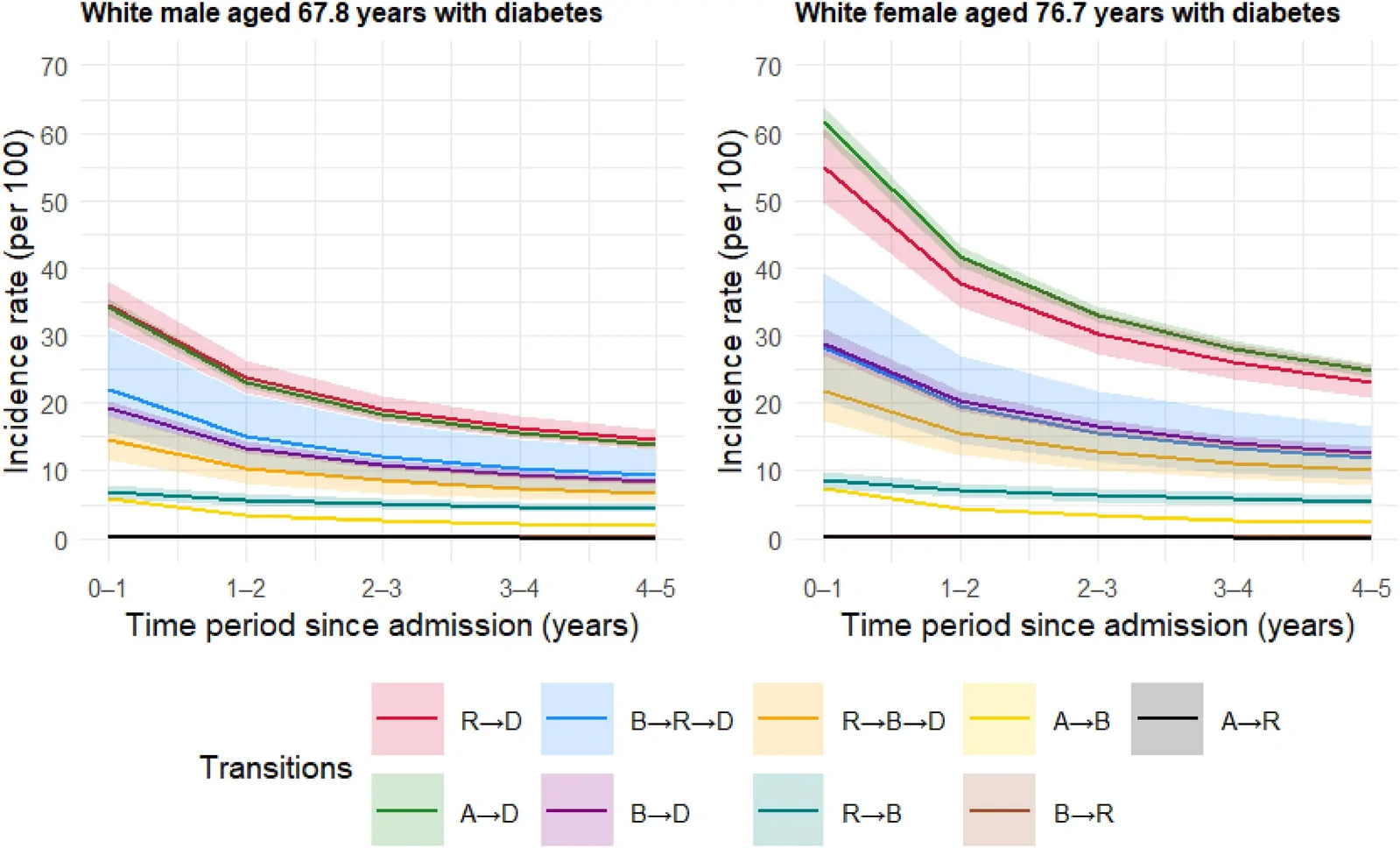
Transition-specific adjusted incidence rates and 95% CIs for a reference white male aged a median age of 67.8 years with diabetes and a reference white female aged a median age of 76.7 years with diabetes. The legend is ordered in descending order of incidence rates column-wise. R, reinfarction; D, death; A, admission for AMI; B, bleeding.

**Figure 5:**
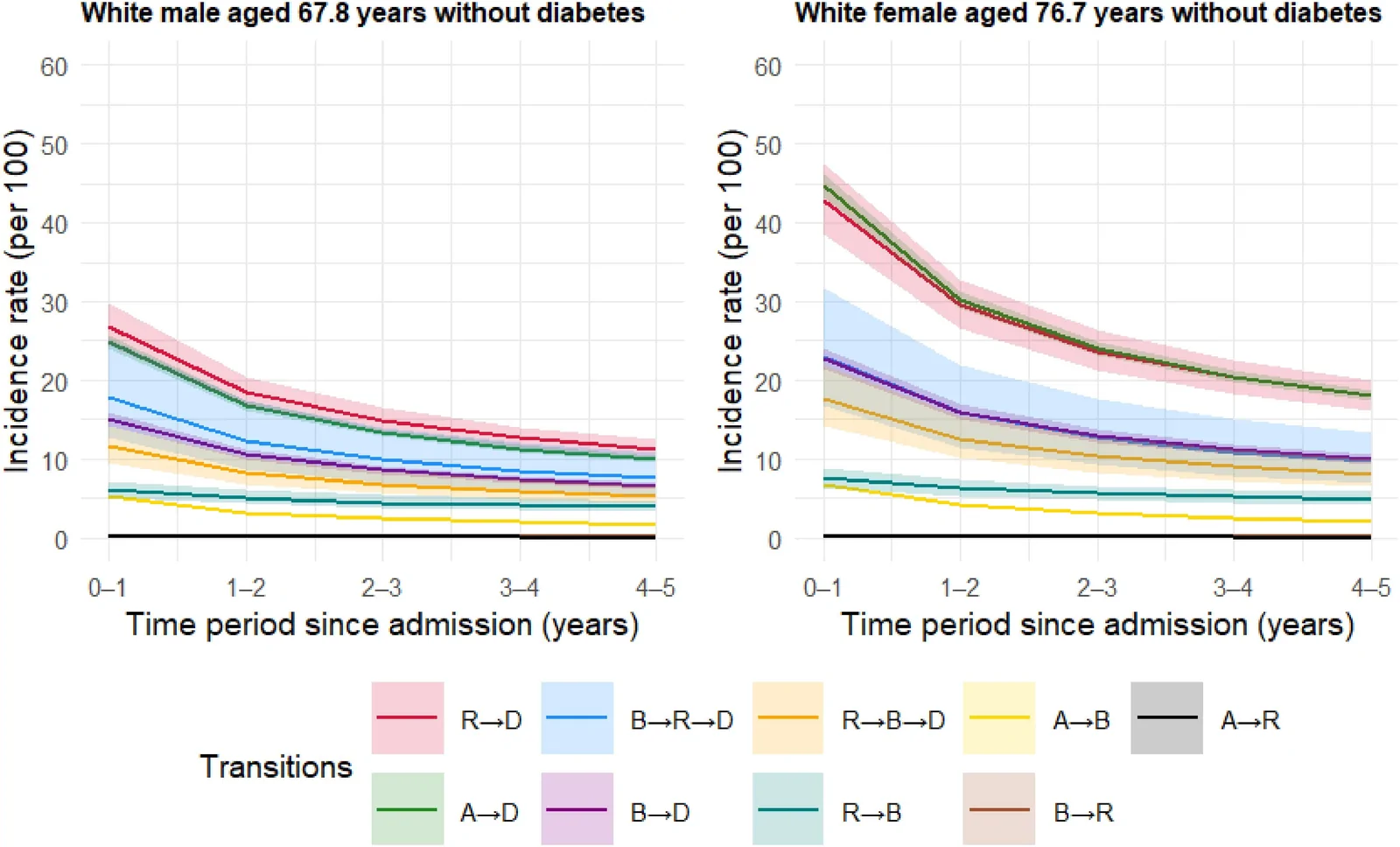
Transition-specific adjusted incidence rates and 95% CIs for a reference white male aged a median age of 67.8 years without diabetes and a reference white female aged a median age of 76.7 years without diabetes. The legend is ordered in descending order of incidence rates column-wise. R, reinfarction; D, death; A, admission for AMI; B, bleeding.

Black patients had lower transition rates than white patients in both sexes. For a reference black female patient with the median age of 71.1 years with diabetes, the incidence rate of transitioning to death after a reinfarction was consistently the highest over the follow-up period and reduced over follow-up time (IR: 44.5; 95% CI 37.8–52.3 ×100PY in Year 1 to IR: 18.7; 95% CI 15.8–22 ×100PY in Year 5). Similarly, for a reference black male patient with a median age of 62.0 years with diabetes, the rate of transitioning to death after a reinfarction was the highest and reduced over follow-up time (IR: 27.7; 95% CI 23.5–32.6 ×100PY in Year 1 to IR: 11.6; 95% CI 9.8–13.7 ×100PY in Year 5) (*Figure 6*). Similar trends were observed in those without diabetes, although with lower rates (*Figure 7*). For patients of the same sex and same age, the rates for bleeding → death, reinfarction → bleeding, reinfarction → death and reinfarction → bleeding → death were higher for black patients than white patients, and, conversely, the rates for AMI admission → bleeding, AMI admission → death, bleeding → reinfarction, and bleeding → reinfarction → death were higher for white than black patients (*Figure 2*).

**Figure 6:**
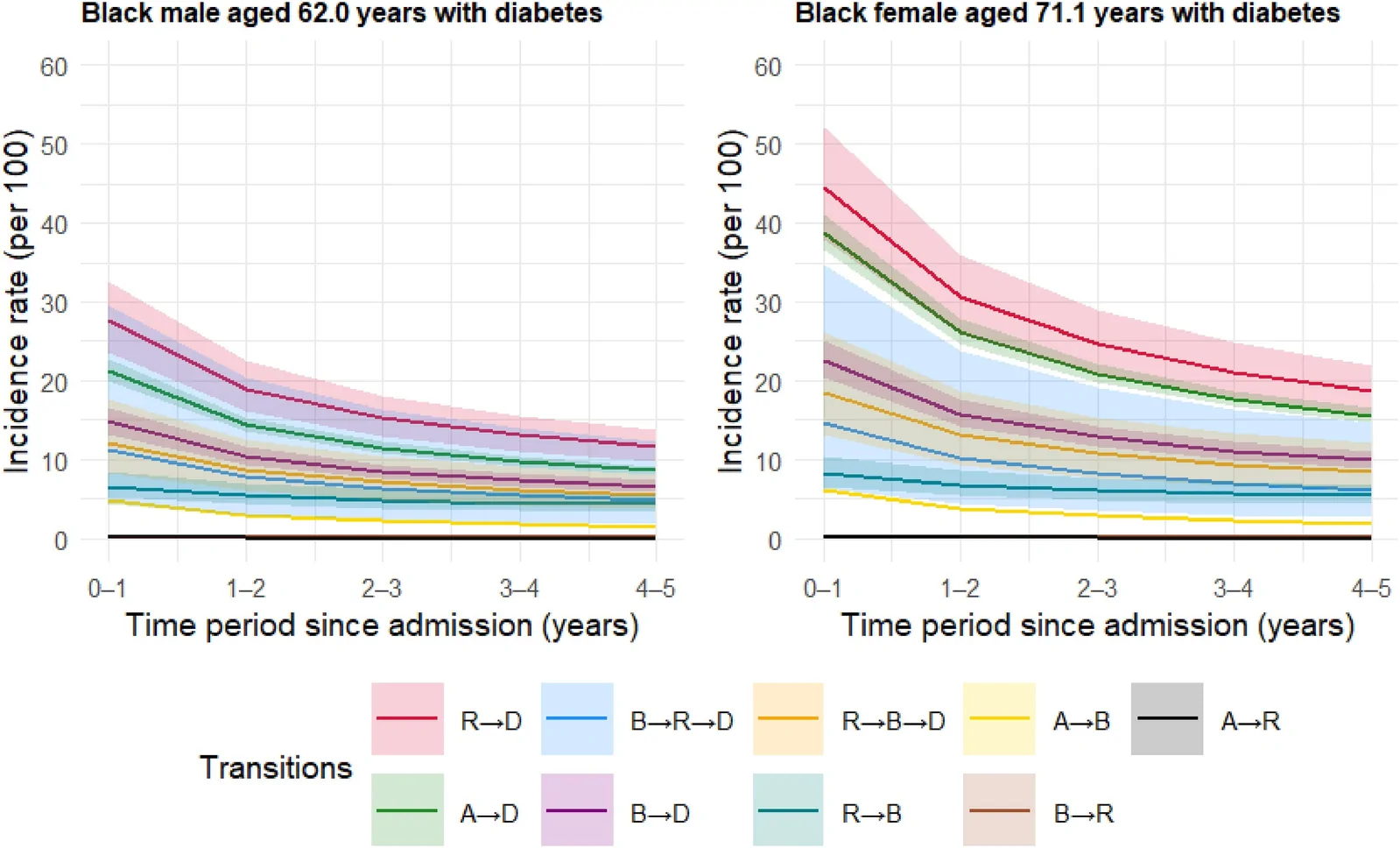
Transition-specific adjusted incidence rates and 95% CIs for a reference black male aged a median age of 62.0 years with diabetes and a reference black female aged a median age of 71.1 years with diabetes. The legend is ordered in descending order of incidence rates column-wise. R, reinfarction; D, death; A, admission for AMI; B, bleeding.

**Figure 7:**
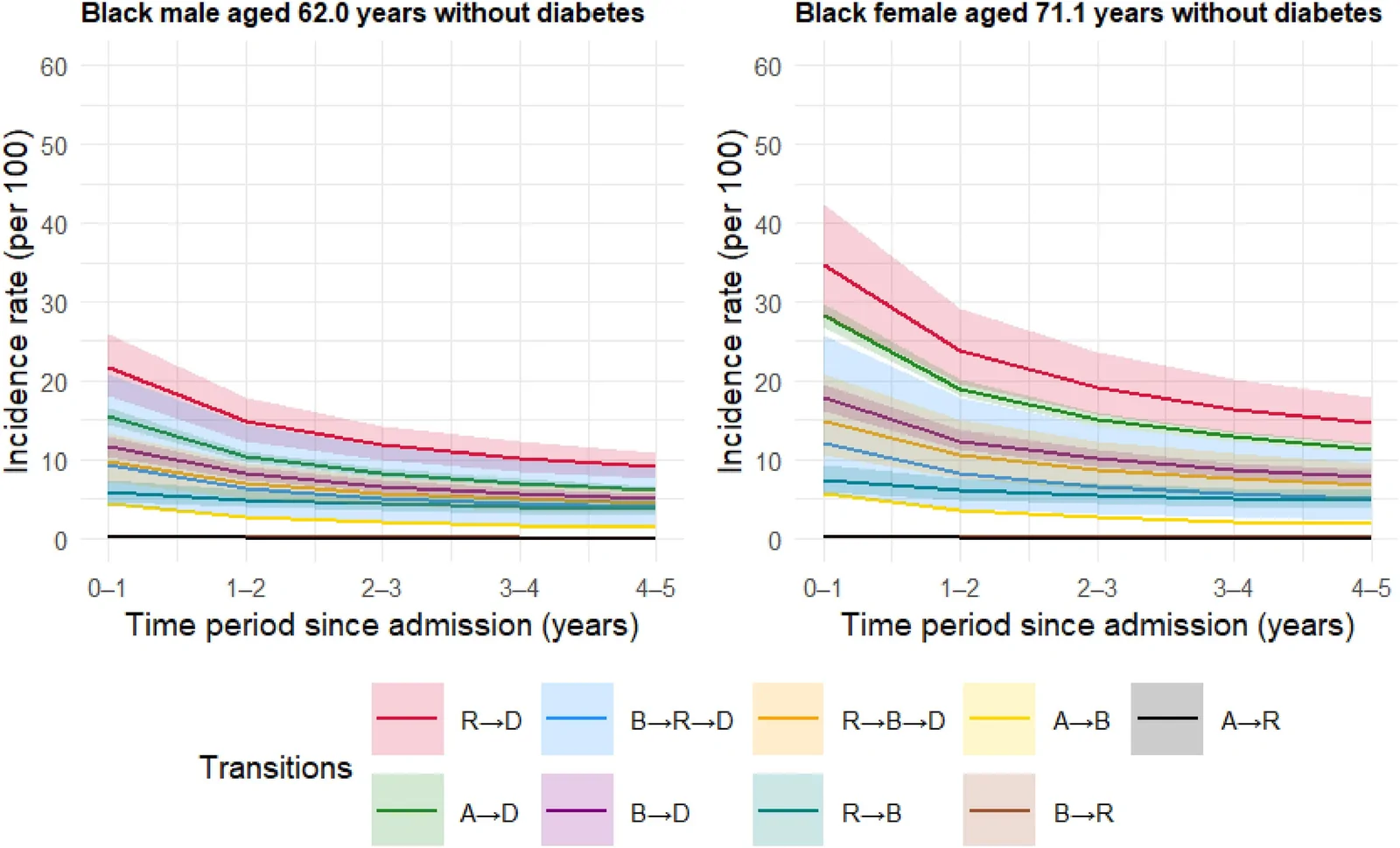
Transition-specific adjusted incidence rates and 95% confidence intervals for a reference black male aged a median age of 62.0 years without diabetes and a reference black female aged a median age of 71.1 years without diabetes. The legend is ordered in descending order of incidence rates column-wise. R, reinfarction; D, death; A, admission for AMI; B, bleeding.

On the other hand, for all races, sexes, and ages, the lowest rates were observed in the transitions to reinfarction either directly or after bleeding, which reduced slightly over follow-up time for both transitions (*Figures 2–7*).

Sensitivity analyses based on three randomly selected imputations (4, 7, and 10) for the Weibull models that were used in estimating the transition rates were consistent with the primary analysis ensuring robustness of the results (see Supplementary material online, *Tables S4*–*S12* for primary analysis and Supplementary material online, *Tables S13*–*S21* for sensitivity analyses).

## Discussion

Our analysis of more than 830 000 patients with AMI provides detailed insights into long-term risks of transitions between major bleeding, reinfarction, and death, using data from the national AMI registry in England and Wales. Our main findings are as follows. (i) Within 5 years after index AMI admission, patients were more likely to transition directly to death (20.6%) and bleeding (19.8%) than to experience a reinfarction (8.1%). (ii) A reference female patient with a median age of 76.3 years with or without diabetes exhibited higher incidence rates for all nine transitions than a reference male patient with a median age of 67.1 years with or without diabetes, with the transition rates decreasing over follow-up time for both sexes. Conversely, the rates for any transition were higher for males than females of the same age. (iii) Direct death and death after reinfarction had the highest transition rates while direct reinfarction or reinfarction after bleeding exhibited the lowest transition rates in both sexes and races throughout the follow-up period. (iv) Transition rates increased with increasing age for both sexes and black/white ethnicities.

To the best of our knowledge, this is the first study to examine transitions between bleeding, reinfarction, and death after AMI admission. Prior studies either analysed these outcomes separately and/or compared how strongly bleeding and reinfarction predict future events without exploring the transition risks between them.^2,7–9^ Understanding how patients move between these events is crucial because they are interrelated and their sequence can influence patient prognosis. This approach can enhance clinical decision-making, inform healthcare planning, support guideline development for secondary prevention, and improve risk stratification in patients with AMI, as it clarifies the sequence and timing of the risks of these events.

Within 5 years of index AMI admission, direct transition to death and bleeding were the most frequent trajectories than reinfarction. This aligns with existing evidence that long-term mortality remains high in AMI populations, particularly in older patients and those with comorbidities, despite the advances in acute care and secondary prevention.^19^ This might reflect a combination of factors, including the ageing population of AMI patients, and the increased burden of multimorbidity. Furthermore, some patients may die from non-cardiovascular causes before experiencing bleeding or reinfarction. Bleeding occurred more frequently than reinfarction, highlighting its growing recognition as a common and serious complication of AMI. This might be attributed to the widespread prolonged use of antiplatelet therapy to prevent ischaemic events, which has been associated with an increased risk of bleeding.^20,21^ The proportions of patients experiencing reinfarction (8%), bleeding (20%), and death (21%) over 5 years reflect the proportion of individuals who had at least one such event during follow-up. Comparisons with existing literature should consider differences in cohort definitions. For example, in a large Danish nationwide study restricted to patients who survived the first year event-free, 5-year risks were 5.5–7.5% for recurrent MI, 2.7–3.9% for bleeding, and 12.5–18.6% for mortality between 2000 and 2017.^22^ Our broader inclusion of all index AMI patients and capture of events across the full 5-year period produce higher proportions that more closely reflect the real-world burden of complications in an unselected national AMI population. Moreover, the bleeding definition, based on HES APC, includes all admissions for clinically significant bleeding, which likely yields higher rates than studies restricted to adjudicated major bleeds.

Our analysis revealed notable sex and racial differences in transition rates for all transitions. Females consistently exhibited higher incidence rates compared with males mainly due to the older age in females than in males, particularly for direct transition to death within the first year without experiencing subsequent events, most notably among white females. However, males and blacks were more likely to die following reinfarction. These align with past studies showing women are at higher risk of both in-hospital and long-term mortality after AMI.^23,24^ A meta-analysis of 35 studies involving 18 555 women and 49 981 men with STEMI who underwent PCI found women had nearly twice the risk for in-hospital all-cause mortality and 1.5 times the risk for 1-year all-cause mortality compared with men. However, this association was significantly reduced after adjusting for baseline cardiovascular risk factors and clinical characteristics.^24^ Higher mortality in women may be due to delayed diagnosis and less aggressive early management, which may increase early mortality risk.^25,26^ Additionally, evidence shows women with acute coronary syndrome often receive suboptimal care, including lower rates of guideline-recommended medical therapies, fewer cardiac catheterizations, and delays in reperfusion.^26,27^ Furthermore, women presenting with AMI tend to be older than men (median age 76.3 vs. 67.1 in our study) and have a higher burden of comorbidities, like hypertension and diabetes which are strong predictors of mortality after AMI. A systematic review evaluating sex differences in long-term mortality after AMI found that higher mortality risk in women is largely explained by differences in age, comorbidities, and treatment, although the extent of this risk varied considerably across studies.^28^ In contrast, men are more likely to undergo early revascularization and survive the initial AMI event but may be at greater risk for reinfarction. According to the American Heart Association’s scientific statement on AMI in women, men are more likely to have a plaque rupture, while women often exhibit plaque erosion, which may influence both initial and recurrent AMI, with plaque rupture associated with increased risk of reinfarction.^25^ Our findings of black patients consistently having higher transition rates for the transitions from reinfarction than white patients align with past studies that found black people at a higher risk of reinfarction.^29,30^ Consequently, they are likely to die from a reinfarction due to delayed early intervention, higher burden of comorbidities, and systemic disparities in post-discharge management.^29,30^ Moreover, prior studies showed that reinfarction is associated with increased risk of death.^9,31^ This may relate to the terminal nature of the reinfarction events but might be because of the evidence that patients with reinfarction are less likely to receive guideline-recommended therapies including aspirin, statins, beta-blockers, P2Y12 inhibitors, and PCI.^9,31^ In our study, P2Y12 inhibitors, beta-blockers, and PCI were used less frequently in patients with reinfarction than in those without. Prior studies showed that the use of evidence-based medications, including beta-blockers, statins, ACEI/ARBs, and antiplatelets, is associated with improved survival in patients with AMI.^32^ Although reinfarctions occurred earlier on average than direct deaths, the instantaneous incidence rate of reinfarction remained lower. This reflects the difference between a modest per-time risk and the cumulative 5-year proportion of patients who eventually experience a reinfarction.

We also found that the transition rates for all transitions in both sexes and races declined over the follow-up period, which is consistent with prior studies that have demonstrated a temporal decline in mortality, bleeding, and reinfarction following AMI, attributed to the increased adoption of evidence-based treatments, bleeding avoidance strategies, and primary and secondary preventive interventions.^7,33^

The findings from this large-scale national analysis underscore the importance of long-term, dynamic risk assessment in patients following AMI. The substantial transition risks from bleeding and reinfarction to death, particularly within the first year, highlight the need for proactive post-discharge monitoring and tailored secondary prevention strategies. The observed disparities by sex, age, diabetes status, and ethnicity suggest that risk stratification should be individualized, incorporating demographic and clinical factors. Multistate modelling offers a powerful framework for clinicians to anticipate patient trajectories and intervene proactively. Integrating such models into decision-support tools could enhance prognostication and guide resource allocation, especially for high-risk groups.

This study provides a first population-level characterization of first-event transitions following AMI within a multistate framework. Building on this work, future studies could extend the model to incorporate recurrent bleeding or reinfarction events to better capture temporal sequences, cumulative burden, and their prognostic impact. Such extensions would require more complex multistate structures with repeated intermediate states and additional assumptions regarding risk dependence over time but may provide further clinically relevant insights into long-term post-AMI trajectories.

Our study offers important strengths while also acknowledging certain limitations that should be considered when interpreting the results. The main strengths of our study include a large, nationally representative cohort with long-term follow-up. Additionally, we employed advanced multistate modelling to characterize transitions between bleeding, reinfarction, and death. This approach provides a more clinically realistic understanding of how patients progress through these adverse outcomes over time, compared with traditional single-endpoint models. Also, we used three linked data registries allowing for more comprehensive capture of patient data and clinical outcomes. However, our study is subject to some limitations. First, due to computational complexities, we used the first imputation for the primary analysis, which might introduce bias if imputations substantially vary across datasets. However, we conducted sensitivity analyses using three more randomly selected imputed datasets, and the results were consistent. Second, our database lacked timing of medication prescriptions; therefore, we modelled them based on whether they were received at any point during the admission, regardless of timing relative to clinical events. Third, our database lacked PCI procedural variables, such as access site and lesion characteristics, and medications like direct oral anticoagulants; therefore, we could not account for these in the analysis. Additionally, left ventricular ejection fraction and Killip classification had high proportions of missing data in the early years (>80%) and therefore were not included in the analysis. Fourth, we were unable to analyse cardiac mortality separately. Fifth, for interpretability, we assumed a constant risk within each transition interval. Although transition durations were short, this assumption may not hold in practice. Sixth, reinfarction events from HES APC could not be subtyped beyond the acute, hospitalized MI as HES APC does not distinguish MI subtypes, although the majority would be Type 1. Seventh, bleeding events could not be mapped to established clinical classifications such as Bleeding Academic Research Consortium (BARC) and may therefore include cases that do not meet BARC 3–5 criteria without significant haemoglobin drop or transfusion. Next, the results may not generalize to other settings with different healthcare systems, demographics, or management practices. Lastly, the possibility of residual unmeasured confounding cannot be excluded, and therefore the results represent associations rather than causality and should not be interpreted in this way.

## Conclusion

This study demonstrates the value of multistate modelling in capturing the dynamic and sequential nature of post-AMI complications, revealing substantial long-term risks of transitioning between major bleeding, reinfarction, and death. Unlike traditional single-endpoint analyses, our approach provides a more nuanced understanding of how patients progress through multiple adverse events over time. The highest transition rates were observed for direct death and death following reinfarction, particularly in older female patients. These findings underscore the need for integrated, patient-centred care strategies and highlight the importance of developing dynamic risk prediction tools that account for interdependent outcomes. Such tools could better inform clinical decision-making and optimize long-term management in diverse patient populations.

## Supplementary Material

zuag088_Supplementary_Data

## Data Availability

Data may be obtained from a third party and are not publicly available. Data analysis methods and codes will be accessible to other researchers for purposes of reproducing the results upon reasonable request to the corresponding author.
