# Supplementary material for: One-year and 5-year transition risks between major bleeding, reinfarction, and death following acute myocardial infarction in England and Wales: a population-based cohort study
Source: Eur Heart J Acute Cardiovasc Care. 2026 Jun 24;15(10):766–77. doi: 10.1093/ehjacc/zuag088 (PMC13717567; doi:10.1093/ehjacc/zuag088)
Supplement: Supplementary file 1 [file zuag088_supplementary_data.docx]

**One-year and 5-year transition risks between major bleeding, reinfarction and death following Acute Myocardial Infarction in England and Wales: A population-based cohort study**

Sharon A. Ayayo, MSc^1^; Glen P. Martin, PhD^2^; Salwa S. Zghebi, PhD^3^; Vicky P. Taxiarchi, PhD^4^; Muhammad Rashid, PhD^5,6^; Prof Evangelos Kontopantelis, PhD^7^; Prof Mamas A. Mamas, DPhil^8,9^

# **Supplementary Figures**

## Supplementary Figure 1: Patient inclusion/Exclusion criteria

## Supplementary Figure 2: 1-year transition-specific adjusted incidence rates for selected covariate scenarios for transitions from admission to bleeding (A) and admission to reinfarction (B)

| A   | B   |
| --- | --- |

## Supplementary Figure 3: 1-year transition-specific adjusted incidence rates for selected covariate scenarios for transitions from admission to death

## Supplementary Figure 4: 1-year transition-specific adjusted incidence rates for selected covariate scenarios for transitions from bleeding to reinfarction (A) and bleeding to death (B)

| A   | B   |
| --- | --- |

**Supplementary Figure 5: 1-year transition-specific adjusted incidence rates for selected covariate scenarios for transitions from bleeding to reinfarction to death**

## Supplementary Figure 6: 1-year transition-specific adjusted incidence rates for selected covariate scenarios for transitions from reinfarction to bleeding (A) and reinfarction to death (B)

| A   | B   |
| --- | --- |

## Supplementary Figure 7: 1-year transition-specific adjusted incidence rates for selected covariate scenarios for transitions from reinfarction to bleeding to death

## Supplementary Figure 8: 5-year transition-specific adjusted incidence rates for selected covariate scenarios for transitions from admission to bleeding (A) and admission to reinfarction (B)

| A   | B   |
| --- | --- |

## Supplementary Figure 9: 5-year transition-specific adjusted incidence rates for selected covariate scenarios for transitions from admission to death

## Supplementary Figure 10: 5-year transition-specific adjusted incidence rates for selected covariate scenarios for transitions from bleeding to reinfarction (A) and bleeding to death (B)

| A   | B   |
| --- | --- |

## Supplementary Figure 11: 5-year transition-specific adjusted incidence rates for selected covariate scenarios for transitions from bleeding to reinfarction to death

## Supplementary Figure 12: 5-year transition-specific adjusted incidence rates for selected covariate scenarios for transitions from reinfarction to bleeding (A) and reinfarction to death (B)

| A   | B   |
| --- | --- |

## Supplementary Figure 13: 5-year transition-specific adjusted incidence rates for selected covariate scenarios for transitions from reinfarction to bleeding to death

# **Supplementary Tables**

## Supplementary Table 1: ICD10 codes for reinfarction and bleeding

| **Reinfarction** | |
| --- | --- |
| I22 | Subsequent Myocardial Infarction |
| **Gastrointestinal bleeding** | |
| I850 | Esophageal varices with bleeding |
| K250 | Gastric ulcer, acute with hemorrhage |
| K252 | Gastric ulcer, acute with both hemorrhage and perforation |
| K254 | Gastric ulcer, chronic or unspecified with hemorrhage |
| K256 | Chronic or unspecified with both hemorrhage and perforation |
| K260 | Duodenal ulcer, acute with hemorrhage |
| K262 | Duodenal ulcer, acute with both hemorrhage and perforation |
| K264 | Duodenal ulcer, chronic or unspecified with hemorrhage |
| K266 | Chronic or unspecified with both hemorrhage and perforation |
| K270 | Peptic ulcer, acute with hemorrhage |
| K272 | Peptic ulcer, acute with both hemorrhage and perforation |
| K274 | Peptic ulcer, chronic or unspecified with hemorrhage |
| K276 | Chronic or unspecified with both hemorrhage and perforation |
| K280 | Gastrojejunal ulcer, acute with hemorrhage |
| K282 | Acute with both hemorrhage and perforation |
| K284 | Gastrojejunal ulcer, chronic or unspecified with hemorrhage |
| K286 | Chronic or unspecified with both hemorrhage and perforation |
| K290 | Acute hemorrhagic gastritis |
| K625 | Hemorrhage of anus and rectum |
| K920 | Hematemesis |
| K921 | Melena |
| K922 | Gastrointestinal hemorrhage, unspecified |
| I841 | Internal hemorrhoids with other complications |
| I843 | External thrombosed hemorrhoids |
| I844 | External hemorrhoids with other complications |
| I848 | Unspecified hemorrhoids with other complications |
| I983 | Esophageal varices with bleeding in diseases classified elsewhere |
| K226 | Gastro-esophageal laceration-hemorrhage syndrome |
| K318 | Angiodysplasia of stomach and duodenum with hemorrhage |
| K552 | Angiodysplasia of the colon with bleeding |
| K558 | Angiodysplasia of the small intestine with hemorrhage |
| K570 | Diverticulosis of the small intestine with perforation, abscess and bleeding |
| K571 | Diverticulosis of the small intestine without perforation and abscess, with bleeding |
| K572 | Diverticulosis of the colon with perforation, abscess and bleeding |
| K573 | Diverticulosis of the colon without perforation or abscess, with bleeding |
| K574 | Diverticular disease of both the small intestine and the large intestine with perforation, abscess and bleeding |
| K575 | Diverticular disease of both the small intestine and the large intestine without perforation or abscess, with bleeding |
| K578 | Diverticular disease of intestine, part unspecified, with perforation, abscess and bleeding |
| K579 | Diverticular disease of intestine, part unspecified, without perforation or abscess, with bleeding |
| **Intracranial bleeding** | |
| I60 | Subarachnoid hemorrhage |
| I600 | Subarachnoid hemorrhage from carotid siphon and bifurcation |
| I601 | Subarachnoid hemorrhage from middle cerebral artery |
| I602 | Subarachnoid hemorrhage from anterior communicating artery |
| I603 | Subarachnoid hemorrhage from posterior communicating artery |
| I604 | Subarachnoid hemorrhage from basilar artery |
| I605 | Subarachnoid hemorrhage from vertebral artery |
| I606 | Subarachnoid hemorrhage from other intracranial arteries |
| I607 | Subarachnoid hemorrhage from intracranial artery, unspecified |
| I608 | Other subarachnoid hemorrhage |
| I609 | Subarachnoid hemorrhage, unspecified |
| I61 | Intracerebral hemorrhage |
| I610 | Intracerebral hemorrhage in hemisphere, subcortical |
| I611 | Intracerebral hemorrhage in hemisphere, cortical |
| I612 | Intracerebral hemorrhage in hemisphere, unspecified |
| I613 | Intracerebral hemorrhage in brain stem |
| I614 | Intracerebral hemorrhage in cerebellum |
| I615 | Intracerebral hemorrhage, intraventricular |
| I616 | Intracerebral hemorrhage, multiple localized |
| I618 | Other intracerebral hemorrhage |
| I619 | Intracerebral hemorrhage, unspecified |
| I62 | Other nontraumatic intracranial hemorrhage |
| I620 | Subdural hemorrhage (acute) (nontraumatic) |
| I621 | Nontraumatic extradural hemorrhage |
| I629 | Intracranial hemorrhage (nontraumatic), unspecified |
| I690 | Sequelae of subarachnoid hemorrhage |
| I691 | Sequelae of intracerebral hemorrhage |
| I692 | Sequelae of other nontraumatic intracranial hemorrhage |
| S063-S065 | Traumatic subdural/epidural hemorrhage |
| S066 | Traumatic subarachnoid hemorrhage |
|  | **Other bleeding** |
| N421 | Congestion and hemorrhage of prostate |
| N02 | Recurrent and persistent hematuria |
| N026 | Recurrent and persistent hematuria, dense deposit disease |
| N028 | Recurrent and persistent hematuria, other |
| N029 | Recurrent and persistent hematuria, unspecified |
| R31 | Unspecified hematuria |
| R310 | Gross hematuria |
| R319 | Hematuria, unspecified |
| M250 | Hemarthrosis |
| R04 | Hemorrhage from respiratory passages |
| R041 | Hemorrhage from throat |
| J942 | Hemothorax |
| R040 | Epistaxis |
| R042 | Hemoptysis |
| R048 | Hemorrhage from other sites in respiratory passages |
| R049 | Hemorrhage from respiratory passages, unspecified |
| I230 | Hemopericardium as current complication following acute myocardial infarction |
| I312 | Hemopericardium, not elsewhere classified |
| S260 | Injury of heart with hemopericardium |
| N836 | Hematosalpinx |
| N857 | Hematometra |
| N897 | Hematocolpos |
| N921 | Excessive and frequent menstruation with irregular cycle |
| N93 | Other abnormal uterine and vaginal bleeding |
| N938 | Other specified abnormal uterine and vaginal bleeding |
| N939 | Abnormal uterine and vaginal bleeding, unspecified |
| N950 | Postmenopausal bleeding |
| I713 | Abdominal aortic aneurysm, ruptured |
| I715 | Thoracoabdominal aortic aneurysm, ruptured |
| K661 | Hemoperitoneum |
| I711 | Thoracic aortic aneurysm, ruptured |
| I718 | Aortic aneurysm of unspecified site, ruptured |

## Supplementary Table 2: Number and percentage of missing data across all variables per AMI admission year

| **Variable** | **2005** | **2006** | **2007** | **2008** | **2009** | **2010** | **2011** | **2012** |
| --- | --- | --- | --- | --- | --- | --- | --- | --- |
| N | 62198 | 57453 | 56430 | 58701 | 61415 | 62588 | 61546 | 61847 |
| Ethnicity | 3404 (5.5%) | 2504 (4.4%) | 1793 (3.2%) | 1449 (2.5%) | 1201 (2.0%) | 1241 (2.0%) | 674 (1.1%) | 664 (1.1%) |
| Previous AMI | 2427 (3.9%) | 2122 (3.7%) | 2945 (5.2%) | 3552 (6.1%) | 4876 (7.9%) | 5049 (8.1%) | 4262 (6.9%) | 4218 (6.8%) |
| Hypertension | 3316 (5.3%) | 2731 (4.8%) | 3363 (6.0%) | 3696 (6.3%) | 4790 (7.8%) | 4812 (7.7%) | 3919 (6.4%) | 4111 (6.6%) |
| Hypercholesterolaemia | 5713 (9.2%) | 5057 (8.8%) | 5329 (9.4%) | 5357 (9.1%) | 6258 (10.2%) | 6058 (9.7%) | 5239 (8.5%) | 4983 (8.1%) |
| Asthma/COPD | 6559 (10.5%) | 6388 (11.1%) | 6597 (11.7%) | 5920 (10.1%) | 6409 (10.4%) | 5460 (8.7%) | 4584 (7.4%) | 4661 (7.5%) |
| Peripheral Vascular Disease | 5379 (8.6%) | 4938 (8.6%) | 6015 (10.7%) | 6794 (11.6%) | 8102 (13.2%) | 6235 (10.0%) | 5210 (8.5%) | 4787 (7.7%) |
| Cerebrovascular Disease | 5128 (8.2%) | 4727 (8.2%) | 4931 (8.7%) | 4944 (8.4%) | 6546 (10.7%) | 5590 (8.9%) | 4611 (7.5%) | 4703 (7.6%) |
| Renal Failure | 5208 (8.4%) | 4565 (7.9%) | 5014 (8.9%) | 5088 (8.7%) | 6487 (10.6%) | 5652 (9.0%) | 4711 (7.7%) | 5007 (8.1%) |
| Smoking Status | 6329 (10.2%) | 5238 (9.1%) | 5153 (9.1%) | 5274 (9.0%) | 5054 (8.2%) | 4472 (7.1%) | 4210 (6.8%) | 3930 (6.4%) |
| Diabetes | 3866 (6.2%) | 2796 (4.9%) | 2230 (4.0%) | 1734 (3.0%) | 2233 (3.6%) | 2567 (4.1%) | 2334 (3.8%) | 2224 (3.6%) |
| Previous PCI | 4770 (7.7%) | 4047 (7.0%) | 4646 (8.2%) | 4745 (8.1%) | 5362 (8.7%) | 5257 (8.4%) | 4599 (7.5%) | 4678 (7.6%) |
| Previous CABG | 4446 (7.1%) | 3852 (6.7%) | 4440 (7.9%) | 4547 (7.7%) | 5201 (8.5%) | 5231 (8.4%) | 4553 (7.4%) | 4593 (7.4%) |
| Previous Angina | 3325 (5.3%) | 3088 (5.4%) | 3691 (6.5%) | 4172 (7.1%) | 5333 (8.7%) | 5455 (8.7%) | 4711 (7.7%) | 4717 (7.6%) |
| Family history of CHD | 33274 (53.5%) | 17046 (29.7%) | 13382 (23.7%) | 13588 (23.1%) | 14448 (23.5%) | 13675 (21.8%) | 13021 (21.2%) | 12325 (19.9%) |
| P2Y12 inhibitors | 10333 (16.6%) | 6916 (12.0%) | 4184 (7.4%) | 3224 (5.5%) | 1555 (2.5%) | 1043 (1.7%) | 257 (0.4%) | 248 (0.4%) |
| Aspirin | 13346 (21.5%) | 13781 (24.0%) | 14013 (24.8%) | 14916 (25.4%) | 15139 (24.7%) | 15464 (24.7%) | 13959 (22.7%) | 14888 (24.1%) |
| Warfarin | 11929 (19.2%) | 9883 (17.2%) | 9001 (16.0%) | 8626 (14.7%) | 9501 (15.5%) | 9226 (14.7%) | 8773 (14.3%) | 9082 (14.7%) |
| Statins | 5533 (8.9%) | 2410 (4.2%) | 1579 (2.8%) | 930 (1.6%) | 815 (1.3%) | 838 (1.3%) | 346 (0.6%) | 350 (0.6%) |
| Glycoprotein 2b/3a inhibitors | 11008 (17.7%) | 8924 (15.5%) | 8212 (14.6%) | 7887 (13.4%) | 8488 (13.8%) | 8346 (13.3%) | 7442 (12.1%) | 8233 (13.3%) |
| PCI | 4218 (6.8%) | 4520 (7.9%) | 8082 (14.3%) | 7850 (13.4%) | 8122 (13.2%) | 9849 (15.7%) | 9761 (15.9%) | 9251 (15.0%) |
| CABG | 5737 (9.2%) | 6315 (11.0%) | 11472 (20.3%) | 10693 (18.2%) | 11328 (18.4%) | 13691 (21.9%) | 13020 (21.2%) | 12706 (20.5%) |
| Heart rate on admission | 8605 (13.8%) | 7609 (13.2%) | 7889 (14.0%) | 8612 (14.7%) | 9166 (14.9%) | 9252 (14.8%) | 9905 (16.1%) | 9484 (15.3%) |
| Systolic blood pressure | 9177 (14.8%) | 7609 (13.2%) | 7612 (13.5%) | 8215 (14.0%) | 8939 (14.6%) | 9050 (14.5%) | 9707 (15.8%) | 9276 (15.0%) |
| Heart failure | 5311 (8.5%) | 4550 (7.9%) | 4949 (8.8%) | 5130 (8.7%) | 6477 (10.5%) | 5630 (9.0%) | 4602 (7.5%) | 4538 (7.3%) |

## Supplementary Table 2 continuation: Number and percentage of missing data across all variables per AMI admission year

| **Variable** | **2013** | **2014** | **2015** | **2016** | **2017** | **2018** | **Total** |
| --- | --- | --- | --- | --- | --- | --- | --- |
| N | 59559 | 58611 | 58114 | 57808 | 60537 | 60577 | 837384 |
| Ethnicity | 728 (1.2%) | 847 (1.4%) | 972 (1.7%) | 1182 (2.0%) | 1607 (2.7%) | 1884 (3.1%) | 20150 (2.4%) |
| Previous AMI | 4212 (7.1%) | 5295 (9.0%) | 5703 (9.8%) | 6075 (10.5%) | 6674 (11.0%) | 6519 (10.8%) | 63929 (7.6%) |
| Hypertension | 4228 (7.1%) | 5245 (8.9%) | 5612 (9.7%) | 5652 (9.8%) | 6294 (10.4%) | 6362 (10.5%) | 64131 (7.7%) |
| Hypercholesterolaemia | 5277 (8.9%) | 6178 (10.5%) | 6283 (10.8%) | 6318 (10.9%) | 7169 (11.8%) | 7303 (12.1%) | 82522 (9.9%) |
| Asthma/COPD | 4544 (7.6%) | 5545 (9.5%) | 6029 (10.4%) | 6411 (11.1%) | 7082 (11.7%) | 7106 (11.7%) | 83295 (9.9%) |
| Peripheral Vascular Disease | 4744 (8.0%) | 5721 (9.8%) | 6161 (10.6%) | 6690 (11.6%) | 7353 (12.1%) | 7317 (12.1%) | 85446 (10.2%) |
| Cerebrovascular Disease | 4622 (7.8%) | 5616 (9.6%) | 6105 (10.5%) | 6608 (11.4%) | 7282 (12.0%) | 7213 (11.9%) | 78626 (9.4%) |
| Renal Failure | 4722 (7.9%) | 5780 (9.9%) | 6359 (10.9%) | 6684 (11.6%) | 7373 (12.2%) | 7329 (12.1%) | 79979 (9.6%) |
| Smoking Status | 3929 (6.6%) | 3788 (6.5%) | 4226 (7.3%) | 4316 (7.5%) | 4347 (7.2%) | 4326 (7.1%) | 64592 (7.7%) |
| Diabetes | 2119 (3.6%) | 1829 (3.1%) | 1415 (2.4%) | 1388 (2.4%) | 1689 (2.8%) | 1771 (2.9%) | 30195 (3.6%) |
| Previous PCI | 4542 (7.6%) | 5898 (10.1%) | 6512 (11.2%) | 6532 (11.3%) | 7236 (12.0%) | 7391 (12.2%) | 76215 (9.1%) |
| Previous CABG | 4468 (7.5%) | 5778 (9.9%) | 6428 (11.1%) | 6490 (11.2%) | 7192 (11.9%) | 7350 (12.1%) | 74569 (8.9%) |
| Previous Angina | 4637 (7.8%) | 5758 (9.8%) | 6194 (10.7%) | 6461 (11.2%) | 7137 (11.8%) | 7271 (12.0%) | 71950 (8.6%) |
| Family history of CHD | 11888 (20.0%) | 13439 (22.9%) | 14919 (25.7%) | 15140 (26.2%) | 14925 (24.7%) | 15367 (25.4%) | 216437 (25.8%) |
| P2Y12 inhibitors | 218 (0.4%) | 205 (0.3%) | 376 (0.6%) | 233 (0.4%) | 263 (0.4%) | 434 (0.7%) | 29489 (3.5%) |
| Aspirin | 7869 (13.2%) | 3779 (6.4%) | 3816 (6.6%) | 3819 (6.6%) | 3951 (6.5%) | 4155 (6.9%) | 142895 (17.1%) |
| Warfarin | 9537 (16.0%) | 11975 (20.4%) | 12919 (22.2%) | 13816 (23.9%) | 14992 (24.8%) | 14856 (24.5%) | 154116 (18.4%) |
| Statins | 364 (0.6%) | 297 (0.5%) | 489 (0.8%) | 476 (0.8%) | 395 (0.7%) | 513 (0.8%) | 15335 (1.8%) |
| Glycoprotein 2b/3a inhibitors | 8879 (14.9%) | 11117 (19.0%) | 11882 (20.4%) | 12618 (21.8%) | 13586 (22.4%) | 13775 (22.7%) | 140397 (16.8%) |
| PCI | 9058 (15.2%) | 10681 (18.2%) | 11089 (19.1%) | 12533 (21.7%) | 14860 (24.5%) | 15131 (25.0%) | 135005 (16.1%) |
| CABG | 12405 (20.8%) | 15125 (25.8%) | 16344 (28.1%) | 18985 (32.8%) | 23785 (39.3%) | 23505 (38.8%) | 195111 (23.3%) |
| Heart rate on admission | 8820 (14.8%) | 6323 (10.8%) | 3080 (5.3%) | 2577 (4.5%) | 2094 (3.5%) | 2169 (3.6%) | 95585 (11.4%) |
| Systolic blood pressure | 8657 (14.5%) | 6133 (10.5%) | 3111 (5.4%) | 2307 (4.0%) | 2129 (3.5%) | 2011 (3.3%) | 93933 (11.2%) |
| Heart failure | 4668 (7.8%) | 5800 (9.9%) | 6244 (10.7%) | 6630 (11.5%) | 7233 (11.9%) | 7246 (12.0%) | 79008 (9.4%) |

## Supplementary Table 3: Management strategies stratified by bleeding, reinfarction and all-cause mortality

|  | **Bleeding** | | **Reinfarction** | | **All-cause mortality** | |
| --- | --- | --- | --- | --- | --- | --- |
| **Variable** | **No** | **Yes** | **No** | **Yes** | **No** | **Yes** |
| N | 658455 | 178929 | 765375 | 72009 | 565417 | 271967 |
| P2Y12 inhibitors | 544935 (82.8%) | 149580 (83.6%) | 639133 (83.5%) | 55382 (76.9%) | 487196 (86.2%) | 207319 (76.2%) |
| Aspirin | 488265 (74.2%) | 133108 (74.4%) | 567412 (74.1%) | 53961 (74.9%) | 453553 (80.2%) | 167820 (61.7%) |
| Warfarin | 25383 (3.9%) | 10867 (6.1%) | 31673 (4.1%) | 4577 (6.4%) | 17660 (3.1%) | 18590 (6.8%) |
| Statins | 531543 (80.7%) | 148848 (83.2%) | 618395 (80.8%) | 61996 (86.1%) | 484256 (85.6%) | 196135 (72.1%) |
| Glycoprotein 2b/3a inhibitors | 48554 (7.4%) | 10543 (5.9%) | 54883 (7.2%) | 4214 (5.9%) | 48762 (8.6%) | 10335 (3.8%) |
| PCI | 320510 (48.7%) | 74169 (41.5%) | 370693 (48.4%) | 23986 (33.3%) | 322632 (57.1%) | 72047 (26.5%) |
| CABG | 14210 (2.2%) | 3712 (2.1%) | 16821 (2.2%) | 1101 (1.5%) | 14851 (2.6%) | 3071 (1.1%) |

## Supplementary Table 4: Primary analysis; Results from the Weibull model for transition 1 (Admission->Bleeding) using the 1^st^ imputed dataset

| **Variable** | **Coefficient** | **95% CI** | **P-value** |
| --- | --- | --- | --- |
| Age | 0.029 | (0.0285, 0.0295) | <0.001 |
| Heartrate | 0.0018 | (0.0016, 0.0021) | <0.001 |
| SBP | -0.0018 | (-0.0019, -0.0016) | <0.001 |
| Female | -0.013 | (-0.0236, -0.0023) | 0.02 |
| Black | -0.0167 | (-0.0653, 0.0319) | 0.50 |
| Asian | -0.1758 | (-0.1997, -0.1519) | <0.001 |
| Mixed | -0.0142 | (-0.1032, 0.0748) | 0.76 |
| Other | -0.0874 | (-0.1259, -0.0490) | <0.001 |
| Previous AMI | -0.0012 | (-0.0162, 0.0138) | 0.87 |
| Hypertension | 0.1114 | (0.1009, 0.1219) | <0.001 |
| Hypercholesterolaemia | -0.0073 | (-0.0184, 0.0038) | 0.20 |
| PVD | 0.121 | (0.0990, 0.1430) | <0.001 |
| Cerebrovascular disease | 0.0932 | (0.0767, 0.1098) | <0.001 |
| Renal failure | 0.2594 | (0.2405, 0.2782) | <0.001 |
| Ex smoker | 0.0953 | (0.0829, 0.1076) | <0.001 |
| Current smoker | 0.0715 | (0.0569, 0.0861) | <0.001 |
| Currently non-smoker but smoking history unknown | 0.0416 | (0.0249, 0.0583) | <0.001 |
| Diabetes | 0.0823 | (0.0701, 0.0944) | <0.001 |
| Previous PCI | 0.123 | (0.1038, 0.1422) | <0.001 |
| Previous CABG | 0.0952 | (0.0746, 0.1157) | <0.001 |
| Previous angina | 0.0743 | (0.0618, 0.0869) | <0.001 |
| Family history of CHD | -0.0899 | (-0.1014, -0.0783) | <0.001 |
| P2Y12 | -0.0236 | (-0.0381, -0.0091) | 0.001 |
| Aspirin | -0.2408 | (-0.2574, -0.2242) | <0.001 |
| Warfarin | 0.1566 | (0.1374, 0.1757) | <0.001 |
| Statin | 0.0192 | (0.0036, 0.0348) | 0.02 |
| Glycoprotein inhibitors | -0.0223 | (-0.0414, -0.0032) | 0.02 |
| Asthma/COPD | 0.1897 | (0.1770, 0.2024) | <0.001 |
| PCI | -0.0572 | (-0.0680, -0.0464) | <0.001 |
| CABG | -0.1206 | (-0.1511, -0.0902) | <0.001 |
| Heart failure | 0.0135 | (-0.0075, 0.0346) | 0.21 |
| Constant | -3.6415 | (-3.6924, -3.5906) | <0.001 |
| log(gamma) | -1.2594 | (-1.2639, -1.2549) | <0.001 |

## Supplementary Table 5: Primary analysis; Results from the Weibull model for transition 2 (Admission->Reinfarction) using the 1^st^ imputed dataset

| **Variable** | **Coefficient** | **95% CI** | **P-value** |
| --- | --- | --- | --- |
| Age | 0.0132 | (0.0125, 0.0139) | <0.001 |
| Heartrate | 0.0048 | (0.0044, 0.0051) | <0.001 |
| SBP | 0.0001 | (-0.0002, 0.0003) | 0.67 |
| Female | -0.0311 | (-0.0481, -0.0142) | <0.001 |
| Black | -0.3341 | (-0.4230, -0.2451) | <0.001 |
| Asian | -0.1427 | (-0.1772, -0.1082) | <0.001 |
| Mixed | -0.2188 | (-0.3712, -0.0663) | 0.01 |
| Other | -0.4914 | (-0.5676, -0.4152) | <0.001 |
| Previous AMI | 2.0567 | (2.0382, 2.0753) | <0.001 |
| Hypertension | -0.0248 | (-0.0414, -0.0083) | 0.003 |
| Hypercholesterolaemia | 0.0357 | (0.0189, 0.0526) | <0.001 |
| PVD | 0.1135 | (0.0837, 0.1432) | <0.001 |
| Cerebrovascular disease | 0.0598 | (0.0366, 0.0831) | <0.001 |
| Renal failure | -0.0785 | (-0.1071, -0.0499) | <0.001 |
| Ex smoker | 0.1285 | (0.1086, 0.1483) | <0.001 |
| Current smoker | 0.2179 | (0.1945, 0.2413) | <0.001 |
| Currently non-smoker but smoking history unknown | 0.045 | (0.0179, 0.0720) | 0.001 |
| Diabetes | 0.0849 | (0.0674, 0.1024) | <0.001 |
| Previous PCI | -0.2767 | (-0.2990, -0.2544) | <0.001 |
| Previous CABG | -0.1543 | (-0.1777, -0.1308) | <0.001 |
| Previous angina | 0.4169 | (0.3992, 0.4345) | <0.001 |
| Family history of CHD | 0.0764 | (0.0591, 0.0938) | <0.001 |
| P2Y12 | -0.2782 | (-0.2985, -0.2579) | <0.001 |
| Aspirin | 0.8477 | (0.8142, 0.8813) | <0.001 |
| Warfarin | -0.0572 | (-0.0867, -0.0277) | <0.001 |
| Statin | -0.1137 | (-0.1392, -0.0881) | <0.001 |
| Glycoprotein inhibitors | 0.1771 | (0.1468, 0.2075) | <0.001 |
| Asthma/COPD | -0.0765 | (-0.0967, -0.0563) | <0.001 |
| PCI | -0.2583 | (-0.2757, -0.2410) | <0.001 |
| CABG | -0.486 | (-0.5426, -0.4293) | <0.001 |
| Heart failure | -0.0832 | (-0.1102, -0.0562) | <0.001 |
| Constant | -5.091 | (-5.1728, -5.0092) | <0.001 |
| log(gamma) | -2.1689 | (-2.1759, -2.1619) | <0.001 |

## Supplementary Table 6: Primary analysis; Results from the Weibull model for transition 3 (Admission->Death) using the 1^st^ imputed dataset

| **Variable** | **Coefficient** | **95% CI** | **P-value** |
| --- | --- | --- | --- |
| Age | 0.0683 | (0.0678, 0.0689) | <0.001 |
| Heartrate | 0.0088 | (0.0086, 0.0090) | <0.001 |
| SBP | -0.0085 | (-0.0087, -0.0083) | <0.001 |
| Female | -0.02 | (-0.0302, -0.0098) | <0.001 |
| Black | -0.0802 | (-0.1321, -0.0283) | 0.002 |
| Asian | -0.1281 | (-0.1537, -0.1025) | <0.001 |
| Mixed | -0.048 | (-0.1502, 0.0542) | 0.36 |
| Other | -0.1397 | (-0.1814, -0.0979) | <0.001 |
| Previous AMI | 0.1428 | (0.1292, 0.1564) | <0.001 |
| Hypertension | 0.009 | (-0.0011, 0.0191) | 0.082 |
| Hypercholesterolaemia | -0.103 | (-0.1146, -0.0914) | <0.001 |
| PVD | 0.3237 | (0.3047, 0.3426) | <0.001 |
| Cerebrovascular disease | 0.2982 | (0.2845, 0.3119) | <0.001 |
| Renal failure | 0.3422 | (0.3267, 0.3578) | <0.001 |
| Ex smoker | 0.1027 | (0.0906, 0.1148) | <0.001 |
| Current smoker | 0.325 | (0.3101, 0.3399) | <0.001 |
| Currently non-smoker but smoking history unknown | 0.157 | (0.1421, 0.1718) | <0.001 |
| Diabetes | 0.3205 | (0.3092, 0.3317) | <0.001 |
| Previous PCI | -0.1486 | (-0.1700, -0.1272) | <0.001 |
| Previous CABG | 0.0557 | (0.0354, 0.0759) | <0.001 |
| Previous angina | 0.0143 | (0.0024, 0.0261) | 0.02 |
| Family history of CHD | -0.2993 | (-0.3127, -0.2859) | <0.001 |
| P2Y12 | -0.2444 | (-0.2563, -0.2324) | <0.001 |
| Aspirin | -1.003 | (-1.0156, -0.9904) | <0.001 |
| Warfarin | -0.2105 | (-0.2289, -0.1921) | <0.001 |
| Statin | -0.3498 | (-0.3623, -0.3372) | <0.001 |
| Glycoprotein inhibitors | 0.049 | (0.0262, 0.0718) | <0.001 |
| Asthma/COPD | 0.228 | (0.2162, 0.2398) | <0.001 |
| PCI | -0.3628 | (-0.3739, -0.3517) | <0.001 |
| CABG | -0.9682 | (-1.0082, -0.9281) | <0.001 |
| Heart failure | 0.3094 | (0.2937, 0.3252) | <0.001 |
| Constant | -5.1922 | (-5.2452, -5.1392) | <0.001 |
| log(gamma) | -0.8337 | (-0.8378, -0.8296) | <0.001 |

## Supplementary Table 7: Primary analysis; Results from the Weibull model for transition 4 (Bleeding->Reinfarction) using the 1^st^ imputed dataset

| **Variable** | **Coefficient** | **95% CI** | **P-value** |
| --- | --- | --- | --- |
| Age | 0.0302 | (0.0268, 0.0336) | <0.001 |
| Heartrate | 0.0056 | (0.0042, 0.0070) | <0.001 |
| SBP | 0.0018 | (0.0007, 0.0029) | 0.001 |
| Female | -0.1284 | (-0.1960, -0.0609) | <0.001 |
| Black | -0.6687 | (-1.1091, -0.2284) | 0.003 |
| Asian | 0.0457 | (-0.1129, 0.2044) | 0.57 |
| Mixed | 0.1204 | (-0.4719, 0.7127) | 0.69 |
| Other | -0.6046 | (-0.9706, -0.2387) | 0.001 |
| Previous AMI | 0.4119 | (0.3339, 0.4899) | <0.001 |
| Hypertension | 0.0434 | (-0.0249, 0.1118) | 0.21 |
| Hypercholesterolaemia | -0.0064 | (-0.0765, 0.0636) | 0.86 |
| PVD | 0.237 | (0.1197, 0.3542) | <0.001 |
| Cerebrovascular disease | 0.1467 | (0.0546, 0.2388) | 0.002 |
| Renal failure | 0.1361 | (0.0319, 0.2403) | 0.01 |
| Ex smoker | 0.1154 | (0.0359, 0.1949) | 0.004 |
| Current smoker | 0.2509 | (0.1503, 0.3516) | <0.001 |
| Currently non-smoker but smoking history unknown | 0.0749 | (-0.0311, 0.1808) | 0.17 |
| Diabetes | 0.1653 | (0.0928, 0.2377) | <0.001 |
| Previous PCI | -0.1879 | (-0.2950, -0.0807) | 0.001 |
| Previous CABG | -0.0258 | (-0.1317, 0.0802) | 0.63 |
| Previous angina | 0.5994 | (0.5286, 0.6703) | <0.001 |
| Family history of CHD | 0.0707 | (-0.0043, 0.1457) | 0.07 |
| P2Y12 | -0.5714 | (-0.6489, -0.4938) | <0.001 |
| Aspirin | 0.9361 | (0.7925, 1.0797) | <0.001 |
| Warfarin | -0.1593 | (-0.2739, -0.0447) | 0.006 |
| Statin | -0.068 | (-0.1696, 0.0336) | 0.19 |
| Glycoprotein inhibitors | 0.0964 | (-0.0474, 0.2401) | 0.19 |
| Asthma/COPD | 0.0698 | (-0.0074, 0.1469) | 0.08 |
| PCI | -0.6452 | (-0.7190, -0.5714) | <0.001 |
| CABG | -0.8824 | (-1.1511, -0.6138) | <0.001 |
| Heart failure | 0.0623 | (-0.0505, 0.1751) | 0.28 |
| Constant | -7.4996 | (-7.8655, -7.1337) | <0.001 |
| log(gamma) | -0.643 | (-0.6709, -0.6152) | <0.001 |

## Supplementary Table 8: Primary analysis; Results from the Weibull model for transition 5 (Bleeding->Death) using the 1^st^ imputed dataset

| **Variable** | **Coefficient** | **95% CI** | **P-value** |
| --- | --- | --- | --- |
| Age | 0.0567 | (0.0558, 0.0576) | <0.001 |
| Heartrate | 0.0061 | (0.0057, 0.0064) | <0.001 |
| SBP | -0.0035 | (-0.0038, -0.0033) | <0.001 |
| Female | -0.0938 | (-0.1106, -0.0769) | <0.001 |
| Black | 0.0716 | (-0.0079, 0.1511) | 0.08 |
| Asian | 0.0273 | (-0.0157, 0.0703) | 0.21 |
| Mixed | 0.013 | (-0.1500, 0.1761) | 0.88 |
| Other | -0.0402 | (-0.1079, 0.0276) | 0.25 |
| Previous AMI | 0.1267 | (0.1047, 0.1487) | <0.001 |
| Hypertension | 0.0013 | (-0.0156, 0.0183) | 0.88 |
| Hypercholesterolaemia | -0.1065 | (-0.1250, -0.0879) | <0.001 |
| PVD | 0.2282 | (0.1977, 0.2587) | <0.001 |
| Cerebrovascular disease | 0.2253 | (0.2026, 0.2480) | <0.001 |
| Renal failure | 0.3605 | (0.3363, 0.3847) | <0.001 |
| Ex smoker | 0.0789 | (0.0590, 0.0987) | <0.001 |
| Current smoker | 0.2998 | (0.2745, 0.3252) | <0.001 |
| Currently non-smoker but smoking history unknown | 0.1074 | (0.0820, 0.1327) | <0.001 |
| Diabetes | 0.2413 | (0.2229, 0.2598) | <0.001 |
| Previous PCI | -0.1161 | (-0.1476, -0.0847) | <0.001 |
| Previous CABG | 0.0637 | (0.0333, 0.0941) | <0.001 |
| Previous angina | 0.0009 | (-0.0182, 0.0199) | 0.93 |
| Family history of CHD | -0.2102 | (-0.2319, -0.1886) | <0.001 |
| P2Y12 | -0.1148 | (-0.1364, -0.0933) | <0.001 |
| Aspirin | -0.3713 | (-0.3948, -0.3478) | <0.001 |
| Warfarin | -0.0025 | (-0.0298, 0.0249) | 0.86 |
| Statin | -0.1859 | (-0.2084, -0.1635) | <0.001 |
| Glycoprotein inhibitors | -0.0653 | (-0.1039, -0.0268) | 0.001 |
| Asthma/COPD | 0.1621 | (0.1429, 0.1813) | <0.001 |
| PCI | -0.3097 | (-0.3276, -0.2919) | <0.001 |
| CABG | -0.6168 | (-0.6776, -0.5559) | <0.001 |
| Heart failure | 0.282 | (0.2554, 0.3086) | <0.001 |
| Constant | -5.0204 | (-5.1141, -4.9266) | <0.001 |
| log(gamma) | -0.7193 | (-0.7262, -0.7123) | <0.001 |

## Supplementary Table 9: Primary analysis; Results from the Weibull model for transition 6 (Reinfarction->Bleeding) using the 1^st^ imputed dataset

| **Variable** | **Coefficient** | **95% CI** | **P-value** |
| --- | --- | --- | --- |
| Age | 0.0289 | (0.0271, 0.0308) | <0.001 |
| Heartrate | 0.0013 | (0.0006, 0.0021) | 0.001 |
| SBP | -0.0005 | (-0.0011, 0.0001) | 0.11 |
| Female | -0.0392 | (-0.0780, -0.0005) | 0.05 |
| Black | 0.1304 | (-0.0664, 0.3271) | 0.194 |
| Asian | -0.1549 | (-0.2399, -0.0699) | <0.001 |
| Mixed | -0.0246 | (-0.3774, 0.3283) | 0.89 |
| Other | 0.0097 | (-0.1670, 0.1865) | 0.91 |
| Previous AMI | -0.0793 | (-0.1183, -0.0403) | <0.001 |
| Hypertension | 0.0885 | (0.0509, 0.1262) | <0.001 |
| Hypercholesterolaemia | -0.0267 | (-0.0644, 0.0109) | 0.16 |
| PVD | 0.0745 | (0.0090, 0.1400) | 0.03 |
| Cerebrovascular disease | 0.1148 | (0.0644, 0.1652) | <0.001 |
| Renal failure | 0.3157 | (0.2549, 0.3766) | <0.001 |
| Ex smoker | 0.0762 | (0.0317, 0.1208) | 0.001 |
| Current smoker | 0.0819 | (0.0265, 0.1374) | 0.004 |
| Currently non-smoker but smoking history unknown | 0.0338 | (-0.0276, 0.0953) | 0.28 |
| Diabetes | 0.1154 | (0.0766, 0.1542) | <0.001 |
| Previous PCI | 0.0455 | (-0.0028, 0.0938) | 0.07 |
| Previous CABG | 0.1945 | (0.1467, 0.2422) | <0.001 |
| Previous angina | 0.1085 | (0.0708, 0.1462) | <0.001 |
| Family history of CHD | -0.0762 | (-0.1159, -0.0366) | <0.001 |
| P2Y12 | 0.0286 | (-0.0190, 0.0762) | 0.24 |
| Aspirin | -0.0985 | (-0.1813, -0.0158) | 0.02 |
| Warfarin | 0.2244 | (0.1640, 0.2849) | <0.001 |
| Statin | -0.038 | (-0.0991, 0.0230) | 0.22 |
| Glycoprotein inhibitors | -0.0581 | (-0.1310, 0.0148) | 0.118 |
| Asthma/COPD | 0.2255 | (0.1818, 0.2693) | <0.001 |
| PCI | -0.1398 | (-0.1792, -0.1004) | <0.001 |
| CABG | -0.1339 | (-0.2623, -0.0054) | 0.04 |
| Heart failure | 0.0567 | (-0.0038, 0.1172) | 0.07 |
| Constant | -4.4517 | (-4.6539, -4.2495) | <0.001 |
| log(gamma) | -0.3098 | (-0.3248, -0.2949) | <0.001 |

## Supplementary Table 10: Primary analysis; Results from the Weibull model for transition 7 (Reinfarction->Death) using the 1^st^ imputed dataset

| **Variable** | **Coefficient** | **95% CI** | **P-value** |
| --- | --- | --- | --- |
| Age | 0.0535 | (0.0521, 0.0549) | <0.001 |
| Heartrate | 0.0056 | (0.0051, 0.0061) | <0.001 |
| SBP | -0.0049 | (-0.0053, -0.0045) | <0.001 |
| Female | -0.0127 | (-0.0387, 0.0133) | 0.34 |
| Black | 0.0895 | (-0.0661, 0.2452) | 0.26 |
| Asian | -0.0415 | (-0.1050, 0.0220) | 0.2 |
| Mixed | -0.2134 | (-0.5278, 0.1009) | 0.18 |
| Other | -0.1002 | (-0.2436, 0.0432) | 0.17 |
| Previous AMI | -0.1112 | (-0.1377, -0.0846) | <0.001 |
| Hypertension | 0.0112 | (-0.0145, 0.0369) | 0.39 |
| Hypercholesterolaemia | -0.0949 | (-0.1218, -0.0680) | <0.001 |
| PVD | 0.2814 | (0.2400, 0.3229) | <0.001 |
| Cerebrovascular disease | 0.217 | (0.1849, 0.2490) | <0.001 |
| Renal failure | 0.3559 | (0.3188, 0.3931) | <0.001 |
| Ex smoker | 0.071 | (0.0402, 0.1017) | <0.001 |
| Current smoker | 0.2078 | (0.1684, 0.2473) | <0.001 |
| Currently non-smoker but smoking history unknown | 0.1069 | (0.0670, 0.1467) | <0.001 |
| Diabetes | 0.2481 | (0.2216, 0.2746) | <0.001 |
| Previous PCI | -0.1774 | (-0.2183, -0.1364) | <0.001 |
| Previous CABG | -0.061 | (-0.0994, -0.0225) | 0.002 |
| Previous angina | 0.0252 | (-0.0006, 0.0510) | 0.06 |
| Family history of CHD | -0.1662 | (-0.1965, -0.1359) | <0.001 |
| P2Y12 | -0.1985 | (-0.2283, -0.1687) | <0.001 |
| Aspirin | -0.4598 | (-0.5052, -0.4143) | <0.001 |
| Warfarin | -0.0972 | (-0.1400, -0.0544) | <0.001 |
| Statin | -0.2949 | (-0.3304, -0.2594) | <0.001 |
| Glycoprotein inhibitors | -0.0935 | (-0.1555, -0.0315) | 0.003 |
| Asthma/COPD | 0.2121 | (0.1826, 0.2415) | <0.001 |
| PCI | -0.3953 | (-0.4259, -0.3648) | <0.001 |
| CABG | -0.4878 | (-0.6001, -0.3756) | <0.001 |
| Heart failure | 0.2958 | (0.2608, 0.3308) | <0.001 |
| Constant | -4.1635 | (-4.3092, -4.0177) | <0.001 |
| log(gamma) | -0.7745 | (-0.7850, -0.7640) | <0.001 |

## Supplementary Table 11: Primary analysis; Results from the Weibull model for transition 8 (Bleeding->Reinfarction->Death) using the 1^st^ imputed dataset

| **Variable** | **Coefficient** | **95% CI** | **P-value** |
| --- | --- | --- | --- |
| Age | 0.0448 | (0.0398, 0.0499) | <0.001 |
| Heartrate | 0.0037 | (0.0019, 0.0054) | <0.001 |
| SBP | -0.0024 | (-0.0038, -0.0010) | 0.001 |
| Female | -0.1473 | (-0.2334, -0.0612) | 0.001 |
| Black | -0.402 | (-1.1480, 0.3440) | 0.29 |
| Asian | -0.2511 | (-0.4863, -0.0158) | 0.04 |
| Mixed | 0.041 | (-0.7659, 0.8478) | 0.92 |
| Other | -0.4137 | (-0.9619, 0.1345) | 0.14 |
| Previous AMI | 0.0078 | (-0.0877, 0.1033) | 0.87 |
| Hypertension | 0.0117 | (-0.0752, 0.0986) | 0.79 |
| Hypercholesterolaemia | -0.0676 | (-0.1571, 0.0219) | 0.14 |
| PVD | 0.2234 | (0.0848, 0.3620) | 0.002 |
| Cerebrovascular disease | 0.1953 | (0.0860, 0.3046) | <0.001 |
| Renal failure | 0.4195 | (0.3014, 0.5376) | <0.001 |
| Ex smoker | 0.0304 | (-0.0715, 0.1322) | 0.56 |
| Current smoker | 0.2029 | (0.0663, 0.3394) | 0.004 |
| Currently non-smoker but smoking history unknown | 0.1269 | (-0.0034, 0.2572) | 0.06 |
| Diabetes | 0.2059 | (0.1151, 0.2968) | <0.001 |
| Previous PCI | -0.1816 | (-0.3336, -0.0297) | 0.02 |
| Previous CABG | 0.0017 | (-0.1359, 0.1392) | 0.98 |
| Previous angina | -0.0148 | (-0.1035, 0.0739) | 0.74 |
| Family history of CHD | -0.1399 | (-0.2414, -0.0383) | 0.01 |
| P2Y12 | -0.0806 | (-0.1782, 0.0170) | 0.11 |
| Aspirin | 0.0714 | (-0.1038, 0.2467) | 0.42 |
| Warfarin | 0.1034 | (-0.0331, 0.2399) | 0.14 |
| Statin | -0.1015 | (-0.2229, 0.0199) | 0.10 |
| Glycoprotein inhibitors | -0.0129 | (-0.2211, 0.1954) | 0.90 |
| Asthma/COPD | 0.1731 | (0.0770, 0.2691) | <0.001 |
| PCI | -0.2184 | (-0.3211, -0.1158) | <0.001 |
| CABG | -0.2224 | (-0.6069, 0.1620) | 0.26 |
| Heart failure | 0.1005 | (-0.0307, 0.2317) | 0.13 |
| Constant | -3.9891 | (-4.5178, -3.4603) | <0.001 |
| log(gamma) | -0.7712 | (-0.8058, -0.7365) | <0.001 |

## Supplementary Table 12: Primary analysis; Results from the Weibull model for transition 9 (Reinfarction->Bleeding->Death) using the 1^st^ imputed dataset

| **Variable** | **Coefficient** | **95% CI** | **P-value** |
| --- | --- | --- | --- |
| Age | 0.0498 | (0.0467, 0.0529) | <0.001 |
| Heartrate | 0.0041 | (0.0031, 0.0051) | <0.001 |
| SBP | -0.001 | (-0.0018, -0.0001) | 0.02 |
| Female | -0.0307 | (-0.0830, 0.0216) | 0.25 |
| Black | 0.1123 | (-0.1736, 0.3983) | 0.44 |
| Asian | -0.0293 | (-0.1617, 0.1032) | 0.67 |
| Mixed | 0.2942 | (-0.2136, 0.8021) | 0.26 |
| Other | 0.0523 | (-0.2268, 0.3314) | 0.71 |
| Previous AMI | -0.0037 | (-0.0580, 0.0506) | 0.89 |
| Hypertension | -0.0337 | (-0.0861, 0.0186) | 0.21 |
| Hypercholesterolaemia | -0.0529 | (-0.1061, 0.0003) | 0.05 |
| PVD | 0.2057 | (0.1214, 0.2900) | <0.001 |
| Cerebrovascular disease | 0.1802 | (0.1153, 0.2451) | <0.001 |
| Renal failure | 0.3977 | (0.3246, 0.4707) | <0.001 |
| Ex smoker | 0.0089 | (-0.0523, 0.0700) | 0.78 |
| Current smoker | 0.1948 | (0.1149, 0.2746) | <0.001 |
| Currently non-smoker but smoking history unknown | 0.0191 | (-0.0646, 0.1027) | 0.66 |
| Diabetes | 0.2165 | (0.1635, 0.2696) | <0.001 |
| Previous PCI | -0.2166 | (-0.2926, -0.1407) | <0.001 |
| Previous CABG | 0.0068 | (-0.0617, 0.0753) | 0.85 |
| Previous angina | -0.0059 | (-0.0582, 0.0463) | 0.82 |
| Family history of CHD | -0.1188 | (-0.1787, -0.0589) | <0.001 |
| P2Y12 | -0.0478 | (-0.1134, 0.0178) | 0.15 |
| Aspirin | 0.1431 | (0.0235, 0.2627) | 0.02 |
| Warfarin | 0.0252 | (-0.0533, 0.1037) | 0.53 |
| Statin | -0.1366 | (-0.2178, -0.0555) | 0.001 |
| Glycoprotein inhibitors | -0.0614 | (-0.1810, 0.0581) | 0.31 |
| Asthma/COPD | 0.1599 | (0.1009, 0.2189) | <0.001 |
| PCI | -0.2908 | (-0.3500, -0.2317) | <0.001 |
| CABG | -0.4751 | (-0.7003, -0.2498) | <0.001 |
| Heart failure | 0.178 | (0.1041, 0.2520) | <0.001 |
| Constant | -4.8606 | (-5.1770, -4.5442) | <0.001 |
| log(gamma) | -0.6654 | (-0.6865, -0.6443) | <0.001 |

## Supplementary Table 13: Sensitivity analysis; Results from the Weibull model for transition 1 (Admission->Bleeding) using the 4^th^, 7^th^ and 10^th^ imputed datasets

|  | **Imputation 4** | | | **Imputation 7** | | | **Imputation 10** | | |
| --- | --- | --- | --- | --- | --- | --- | --- | --- | --- |
| **Variable** | **Coefficient** | **95% CI** | **P-value** | **Coefficient** | **95% CI** | **P-value** | **Coefficient** | **95% CI** | **P-value** |
| Age | 0.0291 | (0.0286, 0.0295) | <0.001 | 0.029 | (0.0286, 0.0295) | <0.001 | 0.0290 | (0.0285, 0.0295) | <0.001 |
| Heartrate | 0.0019 | (0.0016, 0.0021) | <0.001 | 0.0019 | (0.0017, 0.0022) | <0.001 | 0.0018 | (0.0016, 0.0021) | <0.001 |
| SBP | -0.0017 | (-0.0019, -0.0016) | <0.001 | -0.0018 | (-0.0020, -0.0016) | <0.001 | -0.0018 | (-0.0019, -0.0016) | <0.001 |
| Female | -0.0137 | (-0.0243, -0.0030) | 0.01 | -0.0135 | (-0.0241, -0.0028) | 0.01 | -0.0126 | (-0.0232, -0.0019) | 0.02 |
| Black | -0.0179 | (-0.0666, 0.0307) | 0.47 | -0.0174 | (-0.0660, 0.0312) | 0.48 | -0.0178 | (-0.0665, 0.0309) | 0.47 |
| Asian | -0.1767 | (-0.2005, -0.1528) | <0.001 | -0.1772 | (-0.2011, -0.1533) | <0.001 | -0.1724 | (-0.1963, -0.1486) | <0.001 |
| Mixed | -0.0238 | (-0.1135, 0.0659) | 0.60 | -0.0161 | (-0.1054, 0.0731) | 0.72 | -0.0207 | (-0.1103, 0.0689) | 0.65 |
| Other | -0.0894 | (-0.1279, -0.0510) | <0.001 | -0.0944 | (-0.1329, -0.0559) | <0.001 | -0.084 | (-0.1224, -0.0456) | <0.001 |
| Previous AMI | -0.0002 | (-0.0153, 0.0148) | 0.97 | 0.0009 | (-0.0141, 0.0159) | 0.91 | -0.0062 | (-0.0212, 0.0088) | 0.42 |
| Hypertension | 0.1145 | (0.1040, 0.1250) | <0.001 | 0.1167 | (0.1061, 0.1272) | <0.001 | 0.1136 | (0.1031, 0.1242) | <0.001 |
| Hypercholesterolaemia | -0.0121 | (-0.0233, -0.0010) | 0.03 | -0.0126 | (-0.0237, -0.0015) | 0.03 | -0.0115 | (-0.0227, -0.0004) | 0.04 |
| PVD | 0.1254 | (0.1034, 0.1473) | <0.001 | 0.1200 | (0.0980, 0.1420) | <0.001 | 0.1180 | (0.0960, 0.1400) | <0.001 |
| Cerebrovascular disease | 0.0931 | (0.0766, 0.1097) | <0.001 | 0.0973 | (0.0807, 0.1138) | <0.001 | 0.1003 | (0.0838, 0.1168) | <0.001 |
| Renal failure | 0.2544 | (0.2356, 0.2732) | <0.001 | 0.2542 | (0.2354, 0.2730) | <0.001 | 0.2618 | (0.2430, 0.2805) | <0.001 |
| Ex smoker | 0.093 | (0.0806, 0.1054) | <0.001 | 0.0956 | (0.0832, 0.1080) | <0.001 | 0.0972 | (0.0848, 0.1096) | <0.001 |
| Current smoker | 0.0734 | (0.0588, 0.0881) | <0.001 | 0.0741 | (0.0594, 0.0888) | <0.001 | 0.0741 | (0.0595, 0.0888) | <0.001 |
| Currently non-smoker but smoking history unknown | 0.0408 | (0.0241, 0.0575) | <0.001 | 0.0443 | (0.0277, 0.0610) | <0.001 | 0.0438 | (0.0271, 0.0605) | <0.001 |
| Diabetes | 0.0823 | (0.0701, 0.0944) | <0.001 | 0.0843 | (0.0722, 0.0965) | <0.001 | 0.0819 | (0.0697, 0.0940) | <0.001 |
| Previous PCI | 0.1184 | (0.0991, 0.1377) | <0.001 | 0.1213 | (0.1021, 0.1406) | <0.001 | 0.1252 | (0.1060, 0.1445) | <0.001 |
| Previous CABG | 0.0999 | (0.0794, 0.1205) | <0.001 | 0.0933 | (0.0727, 0.1139) | <0.001 | 0.0974 | (0.0769, 0.1180) | <0.001 |
| Previous angina | 0.0761 | (0.0635, 0.0887) | <0.001 | 0.0771 | (0.0646, 0.0897) | <0.001 | 0.0761 | (0.0636, 0.0887) | <0.001 |
| Family history of CHD | -0.0802 | (-0.0917, -0.0687) | <0.001 | -0.0829 | (-0.0944, -0.0714) | <0.001 | -0.0854 | (-0.0970, -0.0739) | <0.001 |
| P2Y12 | -0.0224 | (-0.0369, -0.0079) | 0.003 | -0.0236 | (-0.0382, -0.0091) | 0.001 | -0.0239 | (-0.0384, -0.0094) | 0.001 |
| Aspirin | -0.2444 | (-0.2610, -0.2279) | <0.001 | -0.2509 | (-0.2674, -0.2344) | <0.001 | -0.2447 | (-0.2613, -0.2281) | <0.001 |
| Warfarin | 0.1534 | (0.1342, 0.1726) | <0.001 | 0.1491 | (0.1299, 0.1683) | <0.001 | 0.1588 | (0.1397, 0.1780) | <0.001 |
| Statin | 0.0210 | (0.0054, 0.0366) | 0.01 | 0.0252 | (0.0096, 0.0408) | 0.002 | 0.0203 | (0.0047, 0.0359) | 0.01 |
| Glycoprotein inhibitors | -0.0264 | (-0.0455, -0.0073) | 0.01 | -0.0184 | (-0.0374, 0.0007) | 0.06 | -0.0202 | (-0.0392, -0.0011) | 0.04 |
| Asthma/COPD | 0.1869 | (0.1742, 0.1996) | <0.001 | 0.1849 | (0.1722, 0.1977) | <0.001 | 0.1876 | (0.1749, 0.2003) | <0.001 |
| PCI | -0.0559 | (-0.0668, -0.0451) | <0.001 | -0.0566 | (-0.0674, -0.0457) | <0.001 | -0.0573 | (-0.0681, -0.0464) | <0.001 |
| CABG | -0.1070 | (-0.1374, -0.0766) | <0.001 | -0.1189 | (-0.1494, -0.0884) | <0.001 | -0.1095 | (-0.1399, -0.0792) | <0.001 |
| Heart failure | 0.0209 | (-0.0001, 0.0419) | 0.05 | 0.0111 | (-0.0100, 0.0321) | 0.30 | 0.0146 | (-0.0064, 0.0356) | 0.17 |
| Constant | -3.6544 | (-3.7053, -3.6034) | <0.001 | -3.6472 | (-3.6982, -3.5963) | <0.001 | -3.6384 | (-3.6893, -3.5874) | <0.001 |
| log(gamma) | -1.2594 | (-1.2639, -1.2549) | <0.001 | -1.2592 | (-1.2637, -1.2547) | <0.001 | -1.2593 | (-1.2638, -1.2548) | <0.001 |

## Supplementary Table 14: Sensitivity analysis; Results from the Weibull model for transition 2 (Admission->Reinfarction) using the 4^th^, 7^th^ and 10^th^ imputed datasets

|  | **Imputation 4** | | | **Imputation 7** | | | **Imputation 10** | | |
| --- | --- | --- | --- | --- | --- | --- | --- | --- | --- |
| **Variable** | **Coefficient** | **95% CI** | **P-value** | **Coefficient** | **95% CI** | **P-value** | **Coefficient** | **95% CI** | **P-value** |
| Age | 0.0132 | (0.0124, 0.0139) | <0.001 | 0.0132 | (0.0125, 0.0140) | <0.001 | 0.0132 | (0.0125, 0.0140) | <0.001 |
| Heartrate | 0.0049 | (0.0046, 0.0052) | <0.001 | 0.0049 | (0.0045, 0.0052) | <0.001 | 0.0050 | (0.0047, 0.0053) | <0.001 |
| SBP | 0.0001 | (-0.0002, 0.0003) | 0.67 | 0.0001 | (-0.0002, 0.0004) | 0.50 | 0.0001 | (-0.0002, 0.0003) | 0.85 |
| Female | -0.0328 | (-0.0497, -0.0158) | <0.001 | -0.0310 | (-0.0479, -0.0140) | <0.001 | -0.0331 | (-0.0500, -0.0161) | <0.001 |
| Black | -0.3513 | (-0.4408, -0.2618) | <0.001 | -0.3454 | (-0.4348, -0.2561) | <0.001 | -0.3161 | (-0.4046, -0.2276) | <0.001 |
| Asian | -0.1484 | (-0.1829, -0.1139) | <0.001 | -0.1418 | (-0.1763, -0.1073) | <0.001 | -0.1452 | (-0.1798, -0.1107) | <0.001 |
| Mixed | -0.2194 | (-0.3714, -0.0674) | 0.01 | -0.2169 | (-0.3693, -0.0644) | 0.01 | -0.2274 | (-0.3798, -0.0749) | 0.003 |
| Other | -0.4957 | (-0.5719, -0.4195) | <0.001 | -0.4888 | (-0.5650, -0.4126) | <0.001 | -0.4916 | (-0.5680, -0.4152) | <0.001 |
| Previous AMI | 2.0531 | (2.0346, 2.0717) | <0.001 | 2.0585 | (2.0399, 2.0770) | <0.001 | 2.0543 | (2.0357, 2.0729) | <0.001 |
| Hypertension | -0.0219 | (-0.0384, -0.0054) | 0.01 | -0.0267 | (-0.0432, -0.0101) | 0.002 | -0.0244 | (-0.0409, -0.0078) | 0.004 |
| Hypercholesterolaemia | 0.0304 | (0.0135, 0.0472) | <0.001 | 0.0314 | (0.0145, 0.0482) | <0.001 | 0.0291 | (0.0122, 0.0459) | 0.001 |
| PVD | 0.1132 | (0.0835, 0.1429) | <0.001 | 0.1062 | (0.0765, 0.1359) | <0.001 | 0.1036 | (0.0738, 0.1334) | <0.001 |
| Cerebrovascular disease | 0.0604 | (0.0371, 0.0837) | <0.001 | 0.0622 | (0.0390, 0.0855) | <0.001 | 0.0559 | (0.0326, 0.0792) | <0.001 |
| Renal failure | -0.0735 | (-0.1021, -0.0449) | <0.001 | -0.0867 | (-0.1154, -0.0581) | <0.001 | -0.0806 | (-0.1093, -0.0520) | <0.001 |
| Ex smoker | 0.1206 | (0.1008, 0.1404) | <0.001 | 0.1252 | (0.1054, 0.1451) | <0.001 | 0.1265 | (0.1067, 0.1463) | <0.001 |
| Current smoker | 0.2102 | (0.1869, 0.2336) | <0.001 | 0.2193 | (0.1959, 0.2427) | <0.001 | 0.2103 | (0.1870, 0.2337) | <0.001 |
| Currently non-smoker but smoking history unknown | 0.0369 | (0.0098, 0.0640) | 0.01 | 0.039 | (0.0119, 0.0661) | 0.01 | 0.0335 | (0.0064, 0.0606) | 0.016 |
| Diabetes | 0.0840 | (0.0664, 0.1015) | <0.001 | 0.0857 | (0.0682, 0.1033) | <0.001 | 0.0835 | (0.0659, 0.1010) | <0.001 |
| Previous PCI | -0.2642 | (-0.2865, -0.2419) | <0.001 | -0.2692 | (-0.2915, -0.2470) | <0.001 | -0.2662 | (-0.2886, -0.2439) | <0.001 |
| Previous CABG | -0.1542 | (-0.1776, -0.1308) | <0.001 | -0.1522 | (-0.1756, -0.1287) | <0.001 | -0.1498 | (-0.1732, -0.1264) | <0.001 |
| Previous angina | 0.4226 | (0.4050, 0.4402) | <0.001 | 0.4175 | (0.3999, 0.4351) | <0.001 | 0.4176 | (0.4000, 0.4353) | <0.001 |
| Family history of CHD | 0.0779 | (0.0606, 0.0952) | <0.001 | 0.0748 | (0.0574, 0.0921) | <0.001 | 0.0844 | (0.0671, 0.1017) | <0.001 |
| P2Y12 | -0.2734 | (-0.2938, -0.2531) | <0.001 | -0.2742 | (-0.2946, -0.2539) | <0.001 | -0.2756 | (-0.2959, -0.2553) | <0.001 |
| Aspirin | 0.8272 | (0.7938, 0.8605) | <0.001 | 0.8269 | (0.7937, 0.8602) | <0.001 | 0.8269 | (0.7935, 0.8603) | <0.001 |
| Warfarin | -0.0544 | (-0.0838, -0.0250) | <0.001 | -0.0424 | (-0.0717, -0.0132) | 0.004 | -0.0592 | (-0.0887, -0.0298) | <0.001 |
| Statin | -0.1136 | (-0.1391, -0.0880) | <0.001 | -0.1124 | (-0.1379, -0.0868) | <0.001 | -0.1092 | (-0.1348, -0.0836) | <0.001 |
| Glycoprotein inhibitors | 0.1681 | (0.1377, 0.1985) | <0.001 | 0.1721 | (0.1416, 0.2025) | <0.001 | 0.1782 | (0.1479, 0.2085) | <0.001 |
| Asthma/COPD | -0.0818 | (-0.1020, -0.0616) | <0.001 | -0.0773 | (-0.0975, -0.0571) | <0.001 | -0.0774 | (-0.0976, -0.0572) | <0.001 |
| PCI | -0.2496 | (-0.2670, -0.2322) | <0.001 | -0.2503 | (-0.2677, -0.2329) | <0.001 | -0.2538 | (-0.2712, -0.2364) | <0.001 |
| CABG | -0.5140 | (-0.5710, -0.4569) | <0.001 | -0.4726 | (-0.5290, -0.4163) | <0.001 | -0.4999 | (-0.5568, -0.4430) | <0.001 |
| Heart failure | -0.0824 | (-0.1094, -0.0554) | <0.001 | -0.0781 | (-0.1051, -0.0511) | <0.001 | -0.0832 | (-0.1102, -0.0562) | <0.001 |
| Constant | -5.0794 | (-5.1612, -4.9977) | <0.001 | -5.0911 | (-5.1730, -5.0093) | <0.001 | -5.0883 | (-5.1700, -5.0065) | <0.001 |
| log(gamma) | -2.1688 | (-2.1758, -2.1618) | <0.001 | -2.1687 | (-2.1758, -2.1617) | <0.001 | -2.1689 | (-2.1759, -2.1619) | <0.001 |

## Supplementary Table 15: Sensitivity analysis; Results from the Weibull model for transition 3 (Admission->Death) using the 4^th^, 7^th^ and 10^th^ imputed datasets

|  | **Imputation 4** | | | **Imputation 7** | | | **Imputation 10** | | |
| --- | --- | --- | --- | --- | --- | --- | --- | --- | --- |
| **Variable** | **Coefficient** | **95% CI** | **P-value** | **Coefficient** | **95% CI** | **P-value** | **Coefficient** | **95% CI** | **P-value** |
| Age | 0.0683 | (0.0678, 0.0688) | <0.001 | 0.0683 | (0.0678, 0.0688) | <0.001 | 0.0682 | (0.0677, 0.0687) | <0.001 |
| Heartrate | 0.0089 | (0.0087, 0.0091) | <0.001 | 0.0088 | (0.0086, 0.0090) | <0.001 | 0.0089 | (0.0087, 0.0091) | <0.001 |
| SBP | -0.0086 | (-0.0087, -0.0084) | <0.001 | -0.0085 | (-0.0087, -0.0083) | <0.001 | -0.0085 | (-0.0087, -0.0084) | <0.001 |
| Female | -0.0184 | (-0.0286, -0.0082) | <0.001 | -0.0177 | (-0.0279, -0.0075) | 0.001 | -0.0165 | (-0.0267, -0.0063) | 0.001 |
| Black | -0.0647 | (-0.1164, -0.0130) | 0.01 | -0.0649 | (-0.1165, -0.0133) | 0.01 | -0.0591 | (-0.1107, -0.0074) | 0.03 |
| Asian | -0.1248 | (-0.1504, -0.0992) | <0.001 | -0.1227 | (-0.1484, -0.0971) | <0.001 | -0.1176 | (-0.1432, -0.0920) | <0.001 |
| Mixed | 0.0077 | (-0.0932, 0.1086) | 0.88 | -0.0064 | (-0.1080, 0.0953) | 0.90 | -0.0080 | (-0.1097, 0.0937) | 0.878 |
| Other | -0.1502 | (-0.1920, -0.1083) | <0.001 | -0.1567 | (-0.1987, -0.1148) | <0.001 | -0.1383 | (-0.1802, -0.0964) | <0.001 |
| Previous AMI | 0.1474 | (0.1338, 0.1611) | <0.001 | 0.1418 | (0.1282, 0.1554) | <0.001 | 0.1467 | (0.1331, 0.1603) | <0.001 |
| Hypertension | 0.0091 | (-0.0010, 0.0192) | 0.08 | 0.0121 | (0.0020, 0.0222) | 0.02 | 0.0118 | (0.0017, 0.0219) | 0.02 |
| Hypercholesterolaemia | -0.1026 | (-0.1142, -0.0911) | <0.001 | -0.1016 | (-0.1131, -0.0900) | <0.001 | -0.1010 | (-0.1126, -0.0895) | <0.001 |
| PVD | 0.3357 | (0.3168, 0.3547) | <0.001 | 0.3248 | (0.3059, 0.3437) | <0.001 | 0.3181 | (0.2992, 0.3370) | <0.001 |
| Cerebrovascular disease | 0.3020 | (0.2883, 0.3156) | <0.001 | 0.2938 | (0.2801, 0.3075) | <0.001 | 0.3050 | (0.2913, 0.3187) | <0.001 |
| Renal failure | 0.3421 | (0.3265, 0.3576) | <0.001 | 0.3366 | (0.3211, 0.3522) | <0.001 | 0.3402 | (0.3247, 0.3558) | <0.001 |
| Ex smoker | 0.1019 | (0.0898, 0.1141) | <0.001 | 0.1029 | (0.0908, 0.1151) | <0.001 | 0.1067 | (0.0946, 0.1189) | <0.001 |
| Current smoker | 0.3261 | (0.3112, 0.3410) | <0.001 | 0.3273 | (0.3124, 0.3423) | <0.001 | 0.3248 | (0.3099, 0.3397) | <0.001 |
| Currently non-smoker but smoking history unknown | 0.1514 | (0.1365, 0.1663) | <0.001 | 0.1591 | (0.1442, 0.1739) | <0.001 | 0.1573 | (0.1424, 0.1721) | <0.001 |
| Diabetes | 0.3180 | (0.3068, 0.3293) | <0.001 | 0.3178 | (0.3065, 0.3290) | <0.001 | 0.3179 | (0.3066, 0.3291) | <0.001 |
| Previous PCI | -0.1433 | (-0.1647, -0.1219) | <0.001 | -0.1386 | (-0.1599, -0.1172) | <0.001 | -0.1401 | (-0.1615, -0.1187) | <0.001 |
| Previous CABG | 0.0498 | (0.0296, 0.0701) | <0.001 | 0.0527 | (0.0324, 0.0730) | <0.001 | 0.0586 | (0.0383, 0.0788) | <0.001 |
| Previous angina | 0.0134 | (0.0015, 0.0252) | 0.03 | 0.0147 | (0.0029, 0.0265) | 0.02 | 0.0158 | (0.0039, 0.0276) | 0.01 |
| Family history of CHD | -0.3131 | (-0.3265, -0.2997) | <0.001 | -0.3089 | (-0.3223, -0.2956) | <0.001 | -0.3032 | (-0.3166, -0.2898) | <0.001 |
| P2Y12 | -0.2435 | (-0.2555, -0.2315) | <0.001 | -0.2462 | (-0.2581, -0.2342) | <0.001 | -0.2444 | (-0.2564, -0.2324) | <0.001 |
| Aspirin | -1.0058 | (-1.0183, -0.9932) | <0.001 | -1.0035 | (-1.0161, -0.9910) | <0.001 | -0.9995 | (-1.0121, -0.9869) | <0.001 |
| Warfarin | -0.2128 | (-0.2312, -0.1944) | <0.001 | -0.2047 | (-0.2231, -0.1864) | <0.001 | -0.2043 | (-0.2227, -0.1859) | <0.001 |
| Statin | -0.3477 | (-0.3602, -0.3351) | <0.001 | -0.3497 | (-0.3623, -0.3372) | <0.001 | -0.3532 | (-0.3658, -0.3407) | <0.001 |
| Glycoprotein inhibitors | 0.0621 | (0.0394, 0.0848) | <0.001 | 0.0532 | (0.0305, 0.0760) | <0.001 | 0.0388 | (0.0160, 0.0617) | 0.001 |
| Asthma/COPD | 0.2305 | (0.2187, 0.2423) | <0.001 | 0.2355 | (0.2237, 0.2473) | <0.001 | 0.2326 | (0.2208, 0.2444) | <0.001 |
| PCI | -0.3660 | (-0.3771, -0.3549) | <0.001 | -0.3632 | (-0.3743, -0.3521) | <0.001 | -0.3627 | (-0.3739, -0.3516) | <0.001 |
| CABG | -0.9592 | (-0.9993, -0.9191) | <0.001 | -0.9324 | (-0.9722, -0.8926) | <0.001 | -0.9593 | (-0.9993, -0.9194) | <0.001 |
| Heart failure | 0.3131 | (0.2973, 0.3289) | <0.001 | 0.3162 | (0.3004, 0.3320) | <0.001 | 0.3108 | (0.2950, 0.3266) | <0.001 |
| Constant | -5.1877 | (-5.2407, -5.1347) | <0.001 | -5.199 | (-5.2520, -5.1459) | <0.001 | -5.1911 | (-5.2442, -5.1381) | <0.001 |
| log(gamma) | -0.8334 | (-0.8375, -0.8294) | <0.001 | -0.8333 | (-0.8374, -0.8292) | <0.001 | -0.8337 | (-0.8378, -0.8296) | <0.001 |

## Supplementary Table 16: Sensitivity analysis; Results from the Weibull model for transition 4 (Bleeding->Reinfarction) using the 4^th^, 7^th^ and 10^th^ imputed datasets

|  | **Imputation 4** | | | **Imputation 7** | | | **Imputation 10** | | |
| --- | --- | --- | --- | --- | --- | --- | --- | --- | --- |
| **Variable** | **Coefficient** | **95% CI** | **P-value** | **Coefficient** | **95% CI** | **P-value** | **Coefficient** | **95% CI** | **P-value** |
| Age | 0.0301 | (0.0267, 0.0335) | <0.001 | 0.0312 | (0.0278, 0.0346) | <0.001 | 0.0306 | (0.0272, 0.0340) | <0.001 |
| Heartrate | 0.0053 | (0.0039, 0.0067) | <0.001 | 0.0058 | (0.0045, 0.0072) | <0.001 | 0.0053 | (0.0039, 0.0067) | <0.001 |
| SBP | 0.0017 | (0.0007, 0.0028) | 0.002 | 0.0017 | (0.0006, 0.0028) | 0.002 | 0.0023 | (0.0012, 0.0033) | <0.001 |
| Female | -0.1372 | (-0.2047, -0.0696) | <0.001 | -0.1341 | (-0.2016, -0.0665) | <0.001 | -0.1361 | (-0.2037, -0.0686) | <0.001 |
| Black | -0.6811 | (-1.1215, -0.2408) | 0.002 | -0.6294 | (-1.0593, -0.1996) | 0.004 | -0.6835 | (-1.1239, -0.2431) | 0.002 |
| Asian | 0.0391 | (-0.1190, 0.1972) | 0.63 | 0.0627 | (-0.0946, 0.2200) | 0.43 | 0.0545 | (-0.1037, 0.2126) | 0.50 |
| Mixed | 0.1089 | (-0.4834, 0.7012) | 0.72 | 0.1286 | (-0.4636, 0.7209) | 0.67 | 0.1147 | (-0.4776, 0.7069) | 0.70 |
| Other | -0.6451 | (-1.0175, -0.2728) | 0.001 | -0.6378 | (-1.0101, -0.2654) | 0.001 | -0.6286 | (-1.0010, -0.2563) | 0.001 |
| Previous AMI | 0.4145 | (0.3364, 0.4926) | <0.001 | 0.4228 | (0.3446, 0.5010) | <0.001 | 0.4079 | (0.3296, 0.4861) | <0.001 |
| Hypertension | 0.0508 | (-0.0177, 0.1193) | 0.15 | 0.0532 | (-0.0153, 0.1216) | 0.13 | 0.0573 | (-0.0111, 0.1258) | 0.10 |
| Hypercholesterolaemia | 0.0051 | (-0.0648, 0.0751) | 0.89 | 0.0213 | (-0.0486, 0.0912) | 0.55 | -0.0083 | (-0.0783, 0.0617) | 0.82 |
| PVD | 0.3011 | (0.1872, 0.4150) | <0.001 | 0.2812 | (0.1668, 0.3957) | <0.001 | 0.2794 | (0.1648, 0.3940) | <0.001 |
| Cerebrovascular disease | 0.1834 | (0.0924, 0.2744) | <0.001 | 0.1597 | (0.0682, 0.2513) | 0.001 | 0.1824 | (0.0916, 0.2732) | <0.001 |
| Renal failure | 0.1499 | (0.0468, 0.2530) | 0.004 | 0.1316 | (0.0276, 0.2356) | 0.01 | 0.1560 | (0.0534, 0.2587) | 0.003 |
| Ex smoker | 0.0930 | (0.0137, 0.1723) | 0.02 | 0.1228 | (0.0431, 0.2025) | 0.003 | 0.1212 | (0.0417, 0.2007) | 0.003 |
| Current smoker | 0.2362 | (0.1358, 0.3367) | <0.001 | 0.2744 | (0.1740, 0.3747) | <0.001 | 0.2554 | (0.1549, 0.3560) | <0.001 |
| Currently non-smoker but smoking history unknown | 0.0787 | (-0.0268, 0.1842) | 0.14 | 0.0735 | (-0.0330, 0.1800) | 0.18 | 0.0815 | (-0.0246, 0.1876) | 0.13 |
| Diabetes | 0.1714 | (0.0990, 0.2438) | <0.001 | 0.1713 | (0.0989, 0.2437) | <0.001 | 0.1566 | (0.0841, 0.2292) | <0.001 |
| Previous PCI | -0.1863 | (-0.2938, -0.0789) | 0.001 | -0.1703 | (-0.2771, -0.0635) | 0.002 | -0.1691 | (-0.2761, -0.0620) | 0.002 |
| Previous CABG | -0.0724 | (-0.1795, 0.0346) | 0.19 | -0.0301 | (-0.1362, 0.0760) | 0.58 | -0.0422 | (-0.1485, 0.0640) | 0.44 |
| Previous angina | 0.5820 | (0.5111, 0.6530) | <0.001 | 0.5615 | (0.4904, 0.6325) | <0.001 | 0.5693 | (0.4984, 0.6403) | <0.001 |
| Family history of CHD | 0.0775 | (0.0028, 0.1523) | 0.04 | 0.1063 | (0.0319, 0.1807) | 0.005 | 0.1263 | (0.0522, 0.2005) | 0.001 |
| P2Y12 | -0.5795 | (-0.6572, -0.5019) | <0.001 | -0.582 | (-0.6597, -0.5043) | <0.001 | -0.5806 | (-0.6583, -0.5030) | <0.001 |
| Aspirin | 0.9367 | (0.7941, 1.0792) | <0.001 | 0.939 | (0.7965, 1.0815) | <0.001 | 0.9684 | (0.8233, 1.1135) | <0.001 |
| Warfarin | -0.1838 | (-0.2993, -0.0684) | 0.002 | -0.1162 | (-0.2292, -0.0033) | 0.04 | -0.1401 | (-0.2532, -0.0269) | 0.02 |
| Statin | -0.0778 | (-0.1792, 0.0237) | 0.13 | -0.0791 | (-0.1807, 0.0225) | 0.13 | -0.0593 | (-0.1610, 0.0423) | 0.25 |
| Glycoprotein inhibitors | 0.0809 | (-0.0633, 0.2251) | 0.27 | 0.0138 | (-0.1337, 0.1613) | 0.86 | 0.0389 | (-0.1075, 0.1852) | 0.60 |
| Asthma/COPD | 0.0682 | (-0.0090, 0.1455) | 0.08 | 0.0746 | (-0.0025, 0.1517) | 0.06 | 0.0662 | (-0.0111, 0.1434) | 0.09 |
| PCI | -0.6118 | (-0.6851, -0.5384) | <0.001 | -0.6006 | (-0.6738, -0.5275) | <0.001 | -0.6265 | (-0.6999, -0.5531) | <0.001 |
| CABG | -0.8751 | (-1.1391, -0.6111) | <0.001 | -0.9342 | (-1.2076, -0.6608) | <0.001 | -0.8967 | (-1.1629, -0.6304) | <0.001 |
| Heart failure | 0.1179 | (0.0073, 0.2285) | 0.04 | 0.0447 | (-0.0692, 0.1585) | 0.44 | 0.1274 | (0.0171, 0.2377) | 0.02 |
| Constant | -7.4632 | (-7.8288, -7.0977) | <0.001 | -7.6065 | (-7.9727, -7.2403) | <0.001 | -7.6328 | (-8.0000, -7.2655) | <0.001 |
| log(gamma) | -0.6429 | (-0.6707, -0.6150) | <0.001 | -0.6432 | (-0.6710, -0.6153) | <0.001 | -0.6425 | (-0.6704, -0.6147) | <0.001 |

## Supplementary Table 17: Sensitivity analysis; Results from the Weibull model for transition 5 (Bleeding->Death) using the 4^th^, 7^th^ and 10^th^ imputed datasets

|  | **Imputation 4** | | | **Imputation 7** | | | **Imputation 10** | | |
| --- | --- | --- | --- | --- | --- | --- | --- | --- | --- |
| **Variable** | **Coefficient** | **95% CI** | **P-value** | **Coefficient** | **95% CI** | **P-value** | **Coefficient** | **95% CI** | **P-value** |
| Age | 0.0567 | (0.0558, 0.0577) | <0.001 | 0.0567 | (0.0558, 0.0576) | <0.001 | 0.0566 | (0.0557, 0.0575) | <0.001 |
| Heartrate | 0.0061 | (0.0057, 0.0064) | <0.001 | 0.0061 | (0.0058, 0.0065) | <0.001 | 0.0062 | (0.0059, 0.0066) | <0.001 |
| SBP | -0.0036 | (-0.0039, -0.0034) | <0.001 | -0.0036 | (-0.0039, -0.0033) | <0.001 | -0.0036 | (-0.0039, -0.0034) | <0.001 |
| Female | -0.0923 | (-0.1091, -0.0754) | <0.001 | -0.0927 | (-0.1095, -0.0758) | <0.001 | -0.0923 | (-0.1092, -0.0755) | <0.001 |
| Black | 0.0694 | (-0.0103, 0.1491) | 0.09 | 0.0801 | (0.0006, 0.1597) | 0.05 | 0.0679 | (-0.0121, 0.1479) | 0.096 |
| Asian | 0.0326 | (-0.0105, 0.0756) | 0.14 | 0.0287 | (-0.0143, 0.0717) | 0.19 | 0.0370 | (-0.0061, 0.0800) | 0.092 |
| Mixed | 0.0200 | (-0.1448, 0.1848) | 0.81 | 0.0071 | (-0.1571, 0.1713) | 0.93 | -0.0044 | (-0.1692, 0.1603) | 0.958 |
| Other | -0.0426 | (-0.1104, 0.0251) | 0.22 | -0.0477 | (-0.1156, 0.0203) | 0.17 | -0.0344 | (-0.1019, 0.0330) | 0.317 |
| Previous AMI | 0.1241 | (0.1021, 0.1462) | <0.001 | 0.1314 | (0.1093, 0.1534) | <0.001 | 0.1277 | (0.1057, 0.1497) | <0.001 |
| Hypertension | -0.0010 | (-0.0179, 0.0159) | 0.91 | -0.0036 | (-0.0205, 0.0133) | 0.68 | -0.0023 | (-0.0192, 0.0146) | 0.788 |
| Hypercholesterolaemia | -0.1052 | (-0.1238, -0.0867) | <0.001 | -0.0996 | (-0.1181, -0.0810) | <0.001 | -0.0985 | (-0.1170, -0.0800) | <0.001 |
| PVD | 0.2276 | (0.1972, 0.2581) | <0.001 | 0.2231 | (0.1927, 0.2536) | <0.001 | 0.2213 | (0.1908, 0.2518) | <0.001 |
| Cerebrovascular disease | 0.2286 | (0.2059, 0.2513) | <0.001 | 0.2285 | (0.2059, 0.2511) | <0.001 | 0.2281 | (0.2054, 0.2507) | <0.001 |
| Renal failure | 0.3579 | (0.3337, 0.3821) | <0.001 | 0.359 | (0.3348, 0.3831) | <0.001 | 0.3546 | (0.3304, 0.3787) | <0.001 |
| Ex smoker | 0.0872 | (0.0674, 0.1071) | <0.001 | 0.0813 | (0.0614, 0.1012) | <0.001 | 0.0841 | (0.0642, 0.1040) | <0.001 |
| Current smoker | 0.3075 | (0.2821, 0.3329) | <0.001 | 0.2949 | (0.2696, 0.3203) | <0.001 | 0.3017 | (0.2764, 0.3270) | <0.001 |
| Currently non-smoker but smoking history unknown | 0.1223 | (0.0969, 0.1477) | <0.001 | 0.1127 | (0.0874, 0.1381) | <0.001 | 0.1156 | (0.0902, 0.1409) | <0.001 |
| Diabetes | 0.2425 | (0.2240, 0.2609) | <0.001 | 0.2397 | (0.2213, 0.2582) | <0.001 | 0.2381 | (0.2196, 0.2566) | <0.001 |
| Previous PCI | -0.1217 | (-0.1533, -0.0902) | <0.001 | -0.1227 | (-0.1542, -0.0912) | <0.001 | -0.1127 | (-0.1442, -0.0811) | <0.001 |
| Previous CABG | 0.0698 | (0.0395, 0.1001) | <0.001 | 0.0636 | (0.0332, 0.0940) | <0.001 | 0.0604 | (0.0300, 0.0908) | <0.001 |
| Previous angina | 0.0024 | (-0.0167, 0.0214) | 0.81 | -0.0032 | (-0.0222, 0.0158) | 0.74 | 0.0019 | (-0.0171, 0.0210) | 0.842 |
| Family history of CHD | -0.2124 | (-0.2340, -0.1908) | <0.001 | -0.2020 | (-0.2236, -0.1804) | <0.001 | -0.2220 | (-0.2437, -0.2003) | <0.001 |
| P2Y12 | -0.1159 | (-0.1375, -0.0943) | <0.001 | -0.1162 | (-0.1378, -0.0946) | <0.001 | -0.1154 | (-0.1369, -0.0938) | <0.001 |
| Aspirin | -0.3452 | (-0.3688, -0.3217) | <0.001 | -0.354 | (-0.3774, -0.3305) | <0.001 | -0.3636 | (-0.3871, -0.3402) | <0.001 |
| Warfarin | 0.0000 | (-0.0274, 0.0274) | 1.00 | 0.0039 | (-0.0235, 0.0313) | 0.78 | -0.0036 | (-0.0309, 0.0238) | 0.80 |
| Statin | -0.1936 | (-0.2160, -0.1711) | <0.001 | -0.1872 | (-0.2096, -0.1647) | <0.001 | -0.1868 | (-0.2093, -0.1644) | <0.001 |
| Glycoprotein inhibitors | -0.0756 | (-0.1143, -0.0369) | <0.001 | -0.0756 | (-0.1142, -0.0370) | <0.001 | -0.0678 | (-0.1062, -0.0294) | 0.001 |
| Asthma/COPD | 0.1574 | (0.1381, 0.1766) | <0.001 | 0.1586 | (0.1393, 0.1778) | <0.001 | 0.1626 | (0.1433, 0.1818) | <0.001 |
| PCI | -0.2998 | (-0.3177, -0.2820) | <0.001 | -0.3015 | (-0.3194, -0.2837) | <0.001 | -0.3051 | (-0.3229, -0.2872) | <0.001 |
| CABG | -0.5935 | (-0.6531, -0.5338) | <0.001 | -0.6085 | (-0.6691, -0.5479) | <0.001 | -0.6342 | (-0.6947, -0.5737) | <0.001 |
| Heart failure | 0.2884 | (0.2618, 0.3149) | <0.001 | 0.2897 | (0.2631, 0.3163) | <0.001 | 0.2868 | (0.2603, 0.3133) | <0.001 |
| Constant | -5.0297 | (-5.1235, -4.9359) | <0.001 | -5.034 | (-5.1277, -4.9403) | <0.001 | -5.0179 | (-5.1117, -4.9241) | <0.001 |
| log(gamma) | -0.7200 | (-0.7269, -0.7130) | <0.001 | -0.7198 | (-0.7268, -0.7128) | <0.001 | -0.7191 | (-0.7261, -0.7121) | <0.001 |

## Supplementary Table 18: Sensitivity analysis; Results from the Weibull model for transition 6 (Reinfarction->Bleeding) using the 4^th^, 7^th^ and 10^th^ imputed datasets

|  | **Imputation 4** | | | **Imputation 7** | | | **Imputation 10** | | |
| --- | --- | --- | --- | --- | --- | --- | --- | --- | --- |
| **Variable** | **Coefficient** | **95% CI** | **P-value** | **Coefficient** | **95% CI** | **P-value** | **Coefficient** | **95% CI** | **P-value** |
| Age | 0.029 | (0.0271, 0.0308) | <0.001 | 0.0291 | (0.0272, 0.0309) | <0.001 | 0.029 | (0.0271, 0.0308) | <0.001 |
| Heartrate | 0.0015 | (0.0007, 0.0022) | <0.001 | 0.0012 | (0.0004, 0.0019) | 0.002 | 0.0016 | (0.0009, 0.0024) | <0.001 |
| SBP | -0.0006 | (-0.0012, 0.0000) | 0.07 | -0.0006 | (-0.0012, 0.0000) | 0.05 | -0.0007 | (-0.0013, -0.0001) | 0.02 |
| Female | -0.0422 | (-0.0810, -0.0035) | 0.03 | -0.0372 | (-0.0759, 0.0016) | 0.06 | -0.0407 | (-0.0795, -0.0019) | 0.04 |
| Black | 0.1299 | (-0.0668, 0.3267) | 0.20 | 0.1231 | (-0.0736, 0.3199) | 0.22 | 0.1262 | (-0.0696, 0.3221) | 0.21 |
| Asian | -0.1631 | (-0.2481, -0.0780) | <0.001 | -0.165 | (-0.2500, -0.0801) | <0.001 | -0.1568 | (-0.2418, -0.0718) | <0.001 |
| Mixed | -0.0383 | (-0.3911, 0.3145) | 0.83 | -0.0282 | (-0.3809, 0.3246) | 0.88 | -0.0172 | (-0.3700, 0.3356) | 0.92 |
| Other | -0.0029 | (-0.1803, 0.1745) | 0.98 | -0.0074 | (-0.1849, 0.1700) | 0.93 | -0.0087 | (-0.1861, 0.1688) | 0.92 |
| Previous AMI | -0.0887 | (-0.1276, -0.0497) | <0.001 | -0.0783 | (-0.1173, -0.0392) | <0.001 | -0.0735 | (-0.1125, -0.0345) | <0.001 |
| Hypertension | 0.0813 | (0.0437, 0.1190) | <0.001 | 0.0826 | (0.0450, 0.1203) | <0.001 | 0.0914 | (0.0537, 0.1291) | <0.001 |
| Hypercholesterolaemia | -0.0068 | (-0.0444, 0.0308) | 0.72 | -0.0221 | (-0.0597, 0.0156) | 0.25 | -0.0247 | (-0.0623, 0.0130) | 0.20 |
| PVD | 0.1003 | (0.0353, 0.1652) | 0.002 | 0.0901 | (0.0251, 0.1550) | 0.01 | 0.1196 | (0.0549, 0.1843) | <0.001 |
| Cerebrovascular disease | 0.1253 | (0.0749, 0.1757) | <0.001 | 0.1224 | (0.0720, 0.1727) | <0.001 | 0.1206 | (0.0701, 0.1711) | <0.001 |
| Renal failure | 0.3074 | (0.2466, 0.3681) | <0.001 | 0.3245 | (0.2638, 0.3851) | <0.001 | 0.2981 | (0.2372, 0.3590) | <0.001 |
| Ex smoker | 0.0605 | (0.0161, 0.1049) | 0.01 | 0.0693 | (0.0248, 0.1138) | 0.002 | 0.0616 | (0.0171, 0.1060) | 0.01 |
| Current smoker | 0.0677 | (0.0122, 0.1232) | 0.017 | 0.0798 | (0.0243, 0.1353) | 0.01 | 0.0667 | (0.0112, 0.1222) | 0.02 |
| Currently non-smoker but smoking history unknown | 0.0148 | (-0.0468, 0.0763) | 0.64 | 0.0171 | (-0.0446, 0.0788) | 0.59 | 0.0207 | (-0.0409, 0.0823) | 0.51 |
| Diabetes | 0.1199 | (0.0811, 0.1588) | <0.001 | 0.1286 | (0.0898, 0.1673) | <0.001 | 0.1215 | (0.0827, 0.1604) | <0.001 |
| Previous PCI | 0.0394 | (-0.0090, 0.0877) | 0.11 | 0.0605 | (0.0124, 0.1086) | 0.01 | 0.0568 | (0.0086, 0.1050) | 0.02 |
| Previous CABG | 0.1797 | (0.1318, 0.2276) | <0.001 | 0.1829 | (0.1351, 0.2308) | <0.001 | 0.1813 | (0.1335, 0.2291) | <0.001 |
| Previous angina | 0.1086 | (0.0709, 0.1464) | <0.001 | 0.1079 | (0.0701, 0.1456) | <0.001 | 0.1098 | (0.0720, 0.1476) | <0.001 |
| Family history of CHD | -0.0772 | (-0.1168, -0.0376) | <0.001 | -0.0819 | (-0.1216, -0.0422) | <0.001 | -0.0888 | (-0.1285, -0.0491) | <0.001 |
| P2Y12 | 0.0176 | (-0.0298, 0.0651) | 0.47 | 0.0258 | (-0.0217, 0.0734) | 0.29 | 0.0236 | (-0.0240, 0.0712) | 0.33 |
| Aspirin | -0.1203 | (-0.2023, -0.0383) | 0.004 | -0.1158 | (-0.1983, -0.0333) | 0.01 | -0.1078 | (-0.1908, -0.0247) | 0.01 |
| Warfarin | 0.2176 | (0.1573, 0.2779) | <0.001 | 0.2148 | (0.1549, 0.2747) | <0.001 | 0.1985 | (0.1377, 0.2594) | <0.001 |
| Statin | -0.0306 | (-0.0921, 0.0308) | 0.33 | -0.0279 | (-0.0892, 0.0335) | 0.37 | -0.0409 | (-0.1020, 0.0202) | 0.19 |
| Glycoprotein inhibitors | -0.0907 | (-0.1642, -0.0171) | 0.02 | -0.0653 | (-0.1383, 0.0077) | 0.08 | -0.0853 | (-0.1585, -0.0121) | 0.02 |
| Asthma/COPD | 0.2203 | (0.1764, 0.2641) | <0.001 | 0.2205 | (0.1767, 0.2644) | <0.001 | 0.2268 | (0.1832, 0.2705) | <0.001 |
| PCI | -0.1279 | (-0.1672, -0.0885) | <0.001 | -0.1303 | (-0.1697, -0.0909) | <0.001 | -0.1216 | (-0.1610, -0.0822) | <0.001 |
| CABG | -0.1613 | (-0.2918, -0.0308) | 0.02 | -0.1778 | (-0.3089, -0.0468) | 0.01 | -0.157 | (-0.2875, -0.0265) | 0.02 |
| Heart failure | 0.0544 | (-0.0060, 0.1148) | 0.08 | 0.064 | (0.0038, 0.1243) | 0.04 | 0.0431 | (-0.0177, 0.1038) | 0.17 |
| Constant | -4.4176 | (-4.6195, -4.2157) | <0.001 | -4.4282 | (-4.6297, -4.2268) | <0.001 | -4.429 | (-4.6310, -4.2270) | <0.001 |
| log(gamma) | -0.3098 | (-0.3247, -0.2948) | <0.001 | -0.3096 | (-0.3245, -0.2947) | <0.001 | -0.3099 | (-0.3249, -0.2950) | <0.001 |

## Supplementary Table 19: Sensitivity analysis; Results from the Weibull model for transition 7 (Reinfarction->Death) using the 4^th^, 7^th^ and 10^th^ imputed datasets

|  | **Imputation 4** | | | **Imputation 7** | | | **Imputation 10** | | |
| --- | --- | --- | --- | --- | --- | --- | --- | --- | --- |
| **Variable** | **Coefficient** | **95% CI** | **P-value** | **Coefficient** | **95% CI** | **P-value** | **Coefficient** | **95% CI** | **P-value** |
| Age | 0.0539 | (0.0525, 0.0553) | <0.001 | 0.054 | (0.0526, 0.0554) | <0.001 | 0.0541 | (0.0527, 0.0556) | <0.001 |
| Heartrate | 0.0056 | (0.0051, 0.0061) | <0.001 | 0.0056 | (0.0051, 0.0061) | <0.001 | 0.0057 | (0.0052, 0.0062) | <0.001 |
| SBP | -0.005 | (-0.0054, -0.0046) | <0.001 | -0.005 | (-0.0054, -0.0046) | <0.001 | -0.0049 | (-0.0053, -0.0044) | <0.001 |
| Female | -0.0074 | (-0.0334, 0.0186) | 0.58 | -0.008 | (-0.0340, 0.0180) | 0.55 | -0.0093 | (-0.0354, 0.0167) | 0.48 |
| Black | 0.0456 | (-0.1130, 0.2042) | 0.57 | 0.0457 | (-0.1124, 0.2037) | 0.57 | 0.0554 | (-0.0988, 0.2097) | 0.48 |
| Asian | -0.0533 | (-0.1170, 0.0103) | 0.10 | -0.0451 | (-0.1084, 0.0183) | 0.16 | -0.036 | (-0.0997, 0.0278) | 0.27 |
| Mixed | -0.2437 | (-0.5580, 0.0706) | 0.13 | -0.2282 | (-0.5385, 0.0821) | 0.15 | -0.1937 | (-0.5040, 0.1166) | 0.22 |
| Other | -0.0841 | (-0.2256, 0.0575) | 0.24 | -0.1228 | (-0.2665, 0.0210) | 0.09 | -0.1018 | (-0.2455, 0.0420) | 0.17 |
| Previous AMI | -0.1089 | (-0.1354, -0.0824) | <0.001 | -0.1176 | (-0.1441, -0.0911) | <0.001 | -0.1110 | (-0.1375, -0.0845) | <0.001 |
| Hypertension | 0.0013 | (-0.0244, 0.0270) | 0.92 | -0.0005 | (-0.0262, 0.0252) | 0.97 | 0.0025 | (-0.0232, 0.0282) | 0.85 |
| Hypercholesterolaemia | -0.0847 | (-0.1116, -0.0578) | <0.001 | -0.0826 | (-0.1095, -0.0557) | <0.001 | -0.0821 | (-0.1090, -0.0552) | <0.001 |
| PVD | 0.2766 | (0.2352, 0.3181) | <0.001 | 0.2771 | (0.2357, 0.3185) | <0.001 | 0.2975 | (0.2561, 0.3389) | <0.001 |
| Cerebrovascular disease | 0.2192 | (0.1871, 0.2513) | <0.001 | 0.2219 | (0.1898, 0.2539) | <0.001 | 0.224 | (0.1920, 0.2561) | <0.001 |
| Renal failure | 0.346 | (0.3087, 0.3833) | <0.001 | 0.3487 | (0.3114, 0.3860) | <0.001 | 0.3344 | (0.2971, 0.3717) | <0.001 |
| Ex smoker | 0.0753 | (0.0446, 0.1060) | <0.001 | 0.0734 | (0.0426, 0.1042) | <0.001 | 0.0751 | (0.0443, 0.1058) | <0.001 |
| Current smoker | 0.2270 | (0.1874, 0.2667) | <0.001 | 0.2309 | (0.1913, 0.2705) | <0.001 | 0.2365 | (0.1968, 0.2761) | <0.001 |
| Currently non-smoker but smoking history unknown | 0.1022 | (0.0623, 0.1421) | <0.001 | 0.1069 | (0.0670, 0.1468) | <0.001 | 0.1115 | (0.0716, 0.1514) | <0.001 |
| Diabetes | 0.2481 | (0.2216, 0.2746) | <0.001 | 0.2484 | (0.2219, 0.2750) | <0.001 | 0.2475 | (0.2210, 0.2741) | <0.001 |
| Previous PCI | -0.1837 | (-0.2246, -0.1427) | <0.001 | -0.177 | (-0.2178, -0.1362) | <0.001 | -0.1888 | (-0.2300, -0.1477) | <0.001 |
| Previous CABG | -0.0477 | (-0.0860, -0.0093) | 0.02 | -0.0566 | (-0.0951, -0.0181) | 0.004 | -0.0607 | (-0.0990, -0.0223) | 0.002 |
| Previous angina | 0.025 | (-0.0008, 0.0508) | 0.06 | 0.0316 | (0.0058, 0.0574) | 0.02 | 0.0237 | (-0.0021, 0.0496) | 0.07 |
| Family history of CHD | -0.1525 | (-0.1826, -0.1224) | <0.001 | -0.1754 | (-0.2058, -0.1450) | <0.001 | -0.1609 | (-0.1911, -0.1307) | <0.001 |
| P2Y12 | -0.1922 | (-0.2221, -0.1624) | <0.001 | -0.1898 | (-0.2196, -0.1601) | <0.001 | -0.2005 | (-0.2303, -0.1707) | <0.001 |
| Aspirin | -0.4742 | (-0.5196, -0.4289) | <0.001 | -0.4963 | (-0.5414, -0.4513) | <0.001 | -0.5192 | (-0.5642, -0.4742) | <0.001 |
| Warfarin | -0.0848 | (-0.1273, -0.0423) | <0.001 | -0.0773 | (-0.1195, -0.0351) | <0.001 | -0.0981 | (-0.1407, -0.0554) | <0.001 |
| Statin | -0.2907 | (-0.3264, -0.2550) | <0.001 | -0.2733 | (-0.3090, -0.2376) | <0.001 | -0.2805 | (-0.3161, -0.2449) | <0.001 |
| Glycoprotein inhibitors | -0.1033 | (-0.1652, -0.0414) | 0.001 | -0.0852 | (-0.1473, -0.0231) | 0.01 | -0.0772 | (-0.1386, -0.0157) | 0.01 |
| Asthma/COPD | 0.2048 | (0.1753, 0.2343) | <0.001 | 0.2171 | (0.1877, 0.2466) | <0.001 | 0.2025 | (0.1730, 0.2319) | <0.001 |
| PCI | -0.3994 | (-0.4299, -0.3689) | <0.001 | -0.3961 | (-0.4266, -0.3655) | <0.001 | -0.3891 | (-0.4196, -0.3585) | <0.001 |
| CABG | -0.5089 | (-0.6219, -0.3958) | <0.001 | -0.4183 | (-0.5274, -0.3092) | <0.001 | -0.5114 | (-0.6248, -0.3980) | <0.001 |
| Heart failure | 0.2883 | (0.2532, 0.3233) | <0.001 | 0.2959 | (0.2609, 0.3308) | <0.001 | 0.3010 | (0.2661, 0.3360) | <0.001 |
| Constant | -4.1935 | (-4.3396, -4.0474) | <0.001 | -4.1992 | (-4.3447, -4.0537) | <0.001 | -4.2005 | (-4.3463, -4.0548) | <0.001 |
| log(gamma) | -0.7746 | (-0.7851, -0.7641) | <0.001 | -0.7736 | (-0.7841, -0.7631) | <0.001 | -0.7739 | (-0.7844, -0.7634) | <0.001 |

## Supplementary Table 20: Sensitivity analysis; Results from the Weibull model for transition 8 (Bleeding->Reinfarction->Death) using the 4^th^, 7^th^ and 10^th^ imputed datasets

|  | **Imputation 4** | | | **Imputation 7** | | | **Imputation 10** | | |
| --- | --- | --- | --- | --- | --- | --- | --- | --- | --- |
| **Variable** | **Coefficient** | **95% CI** | **P-value** | **Coefficient** | **95% CI** | **P-value** | **Coefficient** | **95% CI** | **P-value** |
| Age | 0.0437 | (0.0387, 0.0487) | <0.001 | 0.0441 | (0.0391, 0.0491) | <0.001 | 0.0443 | (0.0393, 0.0494) | <0.001 |
| Heartrate | 0.0029 | (0.0012, 0.0047) | 0.001 | 0.0036 | (0.0019, 0.0053) | <0.001 | 0.003 | (0.0013, 0.0048) | <0.001 |
| SBP | -0.0021 | (-0.0034, -0.0007) | 0.003 | -0.0018 | (-0.0031, -0.0004) | 0.01 | -0.0022 | (-0.0035, -0.0008) | 0.002 |
| Female | -0.1398 | (-0.2259, -0.0537) | 0.001 | -0.1405 | (-0.2268, -0.0543) | 0.001 | -0.1265 | (-0.2129, -0.0402) | 0.004 |
| Black | -0.3973 | (-1.1433, 0.3486) | 0.30 | -0.3887 | (-1.0876, 0.3103) | 0.28 | -0.4574 | (-1.2047, 0.2899) | 0.23 |
| Asian | -0.2063 | (-0.4405, 0.0280) | 0.08 | -0.2382 | (-0.4696, -0.0068) | 0.04 | -0.2137 | (-0.4478, 0.0204) | 0.07 |
| Mixed | -0.0166 | (-0.8235, 0.7902) | 0.97 | 0.0927 | (-0.7141, 0.8994) | 0.82 | 0.0246 | (-0.7823, 0.8315) | 0.95 |
| Other | -0.4474 | (-1.0174, 0.1225) | 0.12 | -0.4535 | (-1.0233, 0.1163) | 0.12 | -0.4930 | (-1.0629, 0.0768) | 0.09 |
| Previous AMI | -0.0156 | (-0.1115, 0.0803) | 0.75 | 0.0213 | (-0.0742, 0.1169) | 0.66 | 0.0365 | (-0.0588, 0.1319) | 0.45 |
| Hypertension | -0.0109 | (-0.0977, 0.0759) | 0.81 | 0.0057 | (-0.0811, 0.0925) | 0.90 | 0.0105 | (-0.0764, 0.0974) | 0.81 |
| Hypercholesterolaemia | -0.0805 | (-0.1707, 0.0097) | 0.08 | -0.0909 | (-0.1811, -0.0007) | 0.05 | -0.0626 | (-0.1525, 0.0274) | 0.17 |
| PVD | 0.1743 | (0.0367, 0.3118) | 0.01 | 0.1737 | (0.0358, 0.3117) | 0.01 | 0.1874 | (0.0511, 0.3237) | 0.01 |
| Cerebrovascular disease | 0.2041 | (0.0958, 0.3125) | <0.001 | 0.1789 | (0.0699, 0.2880) | 0.001 | 0.1504 | (0.0419, 0.2589) | 0.01 |
| Renal failure | 0.3825 | (0.2643, 0.5007) | <0.001 | 0.4440 | (0.3264, 0.5616) | <0.001 | 0.4054 | (0.2880, 0.5227) | <0.001 |
| Ex smoker | 0.0630 | (-0.0385, 0.1645) | 0.22 | 0.0634 | (-0.0387, 0.1656) | 0.22 | 0.0923 | (-0.0099, 0.1944) | 0.08 |
| Current smoker | 0.1484 | (0.0130, 0.2839) | 0.03 | 0.1741 | (0.0385, 0.3098) | 0.01 | 0.2350 | (0.0989, 0.3711) | 0.001 |
| Currently non-smoker but smoking history unknown | 0.1396 | (0.0095, 0.2697) | 0.04 | 0.1369 | (0.0057, 0.2681) | 0.04 | 0.1708 | (0.0399, 0.3016) | 0.01 |
| Diabetes | 0.2189 | (0.1282, 0.3097) | <0.001 | 0.2113 | (0.1210, 0.3015) | <0.001 | 0.1815 | (0.0899, 0.2730) | <0.001 |
| Previous PCI | -0.2791 | (-0.4341, -0.1241) | <0.001 | -0.2144 | (-0.3649, -0.0638) | 0.01 | -0.2199 | (-0.3721, -0.0676) | 0.01 |
| Previous CABG | -0.0045 | (-0.1435, 0.1344) | 0.95 | 0.0335 | (-0.1035, 0.1705) | 0.63 | 0.0545 | (-0.0817, 0.1907) | 0.43 |
| Previous angina | 0.0111 | (-0.0775, 0.0996) | 0.81 | 0.0048 | (-0.0840, 0.0936) | 0.92 | 0.0018 | (-0.0869, 0.0904) | 0.97 |
| Family history of CHD | -0.1126 | (-0.2150, -0.0101) | 0.03 | -0.1347 | (-0.2358, -0.0337) | 0.01 | -0.1844 | (-0.2866, -0.0823) | <0.001 |
| P2Y12 | -0.0729 | (-0.1708, 0.0251) | 0.15 | -0.0857 | (-0.1832, 0.0119) | 0.09 | -0.0638 | (-0.1619, 0.0342) | 0.20 |
| Aspirin | 0.0075 | (-0.1632, 0.1783) | 0.93 | 0.0639 | (-0.1091, 0.2369) | 0.47 | 0.0455 | (-0.1309, 0.2220) | 0.61 |
| Warfarin | 0.1130 | (-0.0257, 0.2517) | 0.11 | 0.1141 | (-0.0200, 0.2481) | 0.10 | 0.0754 | (-0.0619, 0.2126) | 0.28 |
| Statin | -0.0936 | (-0.2146, 0.0275) | 0.13 | -0.0952 | (-0.2172, 0.0267) | 0.13 | -0.107 | (-0.2286, 0.0147) | 0.09 |
| Glycoprotein inhibitors | 0.0282 | (-0.1766, 0.2329) | 0.79 | -0.0116 | (-0.2218, 0.1986) | 0.91 | 0.0088 | (-0.2035, 0.2211) | 0.94 |
| Asthma/COPD | 0.1742 | (0.0783, 0.2701) | <0.001 | 0.1721 | (0.0763, 0.2680) | <0.001 | 0.2034 | (0.1078, 0.2989) | <0.001 |
| PCI | -0.2168 | (-0.3189, -0.1147) | <0.001 | -0.1933 | (-0.2948, -0.0919) | <0.001 | -0.198 | (-0.2991, -0.0969) | <0.001 |
| CABG | -0.4037 | (-0.7935, -0.0138) | 0.04 | -0.4237 | (-0.8300, -0.0174) | 0.04 | -0.4077 | (-0.8145, -0.0009) | 0.05 |
| Heart failure | 0.0957 | (-0.0338, 0.2252) | 0.15 | 0.1122 | (-0.0197, 0.2440) | 0.10 | 0.1052 | (-0.0237, 0.2341) | 0.11 |
| Constant | -3.8306 | (-4.3574, -3.3039) | <0.001 | -4.0356 | (-4.5635, -3.5076) | <0.001 | -3.9596 | (-4.4870, -3.4322) | <0.001 |
| log(gamma) | -0.7716 | (-0.8063, -0.7369) | <0.001 | -0.7708 | (-0.8055, -0.7361) | <0.001 | -0.7717 | (-0.8064, -0.7370) | <0.001 |

## Supplementary Table 21: Sensitivity analysis; Results from the Weibull model for transition 9 (Reinfarction->Bleeding->Death) using the 4^th^, 7^th^ and 10^th^ imputed datasets

|  | **Imputation 4** | | | **Imputation 7** | | | **Imputation 10** | | |
| --- | --- | --- | --- | --- | --- | --- | --- | --- | --- |
| **Variable** | **Coefficient** | **95% CI** | **P-value** | **Coefficient** | **95% CI** | **P-value** | **Coefficient** | **95% CI** | **P-value** |
| Age | 0.0502 | (0.0471, 0.0532) | <0.001 | 0.0498 | (0.0467, 0.0528) | <0.001 | 0.0498 | (0.0467, 0.0529) | <0.001 |
| Heartrate | 0.0045 | (0.0035, 0.0055) | <0.001 | 0.0042 | (0.0032, 0.0052) | <0.001 | 0.0044 | (0.0034, 0.0054) | <0.001 |
| SBP | -0.0012 | (-0.0020, -0.0004) | 0.004 | -0.0011 | (-0.0019, -0.0003) | 0.01 | -0.0015 | (-0.0024, -0.0007) | <0.001 |
| Female | -0.0411 | (-0.0935, 0.0114) | 0.13 | -0.0374 | (-0.0897, 0.0148) | 0.16 | -0.0393 | (-0.0917, 0.0131) | 0.14 |
| Black | 0.0739 | (-0.2122, 0.3599) | 0.61 | 0.1022 | (-0.1837, 0.3882) | 0.48 | 0.1194 | (-0.1637, 0.4025) | 0.41 |
| Asian | -0.0317 | (-0.1641, 0.1006) | 0.64 | -0.0285 | (-0.1606, 0.1036) | 0.67 | -0.0239 | (-0.1560, 0.1083) | 0.72 |
| Mixed | 0.2482 | (-0.2598, 0.7562) | 0.34 | 0.297 | (-0.2108, 0.8049) | 0.25 | 0.2827 | (-0.2250, 0.7904) | 0.28 |
| Other | 0.0330 | (-0.2489, 0.3149) | 0.82 | 0.0234 | (-0.2585, 0.3053) | 0.87 | 0.0404 | (-0.2417, 0.3225) | 0.78 |
| Previous AMI | -0.0211 | (-0.0752, 0.0331) | 0.45 | -0.005 | (-0.0594, 0.0495) | 0.86 | -0.0137 | (-0.0680, 0.0405) | 0.62 |
| Hypertension | -0.0427 | (-0.0951, 0.0096) | 0.11 | -0.037 | (-0.0893, 0.0153) | 0.17 | -0.0345 | (-0.0867, 0.0178) | 0.20 |
| Hypercholesterolaemia | -0.0474 | (-0.1004, 0.0056) | 0.08 | -0.0542 | (-0.1072, -0.0011) | 0.05 | -0.0609 | (-0.1139, -0.0079) | 0.02 |
| PVD | 0.199 | (0.1156, 0.2823) | <0.001 | 0.2131 | (0.1295, 0.2967) | <0.001 | 0.1997 | (0.1161, 0.2834) | <0.001 |
| Cerebrovascular disease | 0.1653 | (0.1004, 0.2301) | <0.001 | 0.1724 | (0.1077, 0.2372) | <0.001 | 0.1761 | (0.1112, 0.2411) | <0.001 |
| Renal failure | 0.3753 | (0.3022, 0.4483) | <0.001 | 0.368 | (0.2950, 0.4411) | <0.001 | 0.3930 | (0.3199, 0.4661) | <0.001 |
| Ex smoker | 0.0078 | (-0.0533, 0.0688) | 0.80 | 0.0066 | (-0.0544, 0.0677) | 0.83 | -0.0081 | (-0.0691, 0.0529) | 0.79 |
| Current smoker | 0.2014 | (0.1209, 0.2819) | <0.001 | 0.1761 | (0.0960, 0.2562) | <0.001 | 0.1771 | (0.0967, 0.2575) | <0.001 |
| Currently non-smoker but smoking history unknown | 0.040 | (-0.0436, 0.1235) | 0.35 | 0.0227 | (-0.0612, 0.1065) | 0.60 | 0.0142 | (-0.0694, 0.0979) | 0.74 |
| Diabetes | 0.2192 | (0.1661, 0.2723) | <0.001 | 0.2257 | (0.1727, 0.2788) | <0.001 | 0.2147 | (0.1616, 0.2677) | <0.001 |
| Previous PCI | -0.2032 | (-0.2787, -0.1276) | <0.001 | -0.1842 | (-0.2589, -0.1095) | <0.001 | -0.1955 | (-0.2705, -0.1204) | <0.001 |
| Previous CABG | -0.0069 | (-0.0758, 0.0620) | 0.85 | -0.026 | (-0.0948, 0.0428) | 0.46 | -0.0281 | (-0.0970, 0.0407) | 0.42 |
| Previous angina | -0.0007 | (-0.0529, 0.0515) | 0.98 | -0.0059 | (-0.0582, 0.0464) | 0.83 | 0.0025 | (-0.0498, 0.0547) | 0.93 |
| Family history of CHD | -0.0856 | (-0.1450, -0.0262) | 0.01 | -0.1234 | (-0.1834, -0.0634) | <0.001 | -0.1210 | (-0.1809, -0.0611) | <0.001 |
| P2Y12 | -0.0518 | (-0.1168, 0.0133) | 0.12 | -0.0521 | (-0.1174, 0.0133) | 0.12 | -0.0552 | (-0.1207, 0.0102) | 0.10 |
| Aspirin | 0.2190 | (0.0985, 0.3395) | <0.001 | 0.1737 | (0.0544, 0.2931) | 0.004 | 0.1912 | (0.0705, 0.3118) | 0.002 |
| Warfarin | 0.0182 | (-0.0600, 0.0964) | 0.65 | -0.0042 | (-0.0825, 0.0742) | 0.92 | 0.0261 | (-0.0530, 0.1052) | 0.52 |
| Statin | -0.1555 | (-0.2377, -0.0733) | <0.001 | -0.1474 | (-0.2291, -0.0657) | <0.001 | -0.1308 | (-0.2120, -0.0496) | 0.002 |
| Glycoprotein inhibitors | -0.0925 | (-0.2149, 0.0300) | 0.14 | -0.0627 | (-0.1829, 0.0574) | 0.31 | -0.0647 | (-0.1861, 0.0567) | 0.30 |
| Asthma/COPD | 0.1825 | (0.1237, 0.2412) | <0.001 | 0.1825 | (0.1238, 0.2413) | <0.001 | 0.1420 | (0.0831, 0.2010) | <0.001 |
| PCI | -0.3144 | (-0.3733, -0.2555) | <0.001 | -0.3021 | (-0.3613, -0.2430) | <0.001 | -0.2743 | (-0.3333, -0.2152) | <0.001 |
| CABG | -0.5642 | (-0.7998, -0.3285) | <0.001 | -0.5752 | (-0.8126, -0.3378) | <0.001 | -0.5462 | (-0.7771, -0.3154) | <0.001 |
| Heart failure | 0.1943 | (0.1208, 0.2678) | <0.001 | 0.1855 | (0.1117, 0.2593) | <0.001 | 0.2104 | (0.1366, 0.2842) | <0.001 |
| Constant | -4.9283 | (-5.2460, -4.6106) | <0.001 | -4.8511 | (-5.1689, -4.5334) | <0.001 | -4.8272 | (-5.1451, -4.5093) | <0.001 |
| log(gamma) | -0.6647 | (-0.6858, -0.6436) | <0.001 | -0.6647 | (-0.6858, -0.6436) | <0.001 | -0.6647 | (-0.6858, -0.6436) | <0.001 |
